# Immunogenicity, Impact on Carriage and Reactogenicity of 10-Valent Pneumococcal Non-Typeable *Haemophilus influenzae* Protein D Conjugate Vaccine in Kenyan Children Aged 1–4 Years: A Randomized Controlled Trial

**DOI:** 10.1371/journal.pone.0085459

**Published:** 2014-01-21

**Authors:** Laura L. Hammitt, John Ojal, Mahfudh Bashraheil, Susan C. Morpeth, Angela Karani, Ahsan Habib, Dorota Borys, David Goldblatt, J. Anthony G Scott

**Affiliations:** 1 KEMRI-Wellcome Trust Research Programme, Centre for Geographic Medicine-Coast, Kilifi, Kenya; 2 Department of International Health, Johns Hopkins Bloomberg School of Public Health, Baltimore, Maryland, United States of America; 3 Nuffield Department of Clinical Medicine, University of Oxford, Oxford, United Kingdom; 4 GlaxoSmithKline Vaccines, Wavre, Belgium; 5 Institute of Child Health, University College London, London, United Kingdom; 6 Department of Infectious Disease Epidemiology, London School of Hygiene and Tropical Medicine, London, United Kingdom; University of Cambridge, United Kingdom

## Abstract

**Background:**

The impact on carriage and optimal schedule for primary vaccination of older children with 10-valent pneumococcal non-typeable *Haemophilus influenzae* protein-D conjugate vaccine (PHiD-CV) are unknown.

**Methods:**

600 Kenyan children aged 12–59 months were vaccinated at days 0, 60 and 180 in a double-blind randomized controlled trial according to the following vaccine sequence: Group A: PHiD-CV, PHiD-CV, diphtheria/tetanus/acellular pertussis vaccine (DTaP); Group B: PHiD-CV, DTaP, PHiD-CV; Group C: hepatitis A vaccine (HAV), DTaP, HAV. Nasopharyngeal carriage of *Streptococcus pneumoniae* was measured at five timepoints. In 375 subjects, serotype-specific responses were measured by 22F-inhibition ELISA and opsonophagocytic killing assays (OPA) one month after vaccination.

**Results:**

Following one dose of PHiD-CV, >90% of recipients developed IgG≥0.35 µg/mL to serotypes 1, 4, 5, 7F, 9V and 18C and OPA≥8 to serotypes 4, 7F, 9V, 18C, 23F. After a second dose >90% of recipients had IgG≥0.35 µg/mL to all vaccine serotypes and OPA≥8 to all vaccine serotypes except 1 and 6B. At day 180, carriage of vaccine-type pneumococci was 21% in recipients of two doses of PHiD-CV (Group A) compared to 31% in controls (p = 0.04). Fever after dose 1 was reported by 41% of PHiD-CV recipients compared to 26% of HAV recipients (p<0.001). Other local and systemic adverse experiences were similar between groups.

**Conclusions:**

Vaccination of children aged 12–59 months with two doses of PHiD-CV two to six months apart was immunogenic, reduced vaccine-type pneumococcal carriage and was well-tolerated. Administration of PHiD-CV would be expected to provide effective protection against vaccine-type disease.

**Trial Registration:**

ClinicalTrials.gov NCT01028326

## Background

Invasive pneumococcal disease (IPD) is a major cause of morbidity and mortality in Kenya [1], [2]. In countries where pneumococcal conjugate vaccine (PCV) has been introduced into the childhood immunization schedule, it has reduced the incidence of vaccine-type IPD by more than 75% among young children [3], [4]. Reductions in IPD among unvaccinated children and adults have also been achieved through decreased transmission of pneumococcal infection from vaccinated children [3], [5], [6]. The first generation PCV offered protection against seven (4, 6B, 9V, 14, 18C, 19F, and 23F) of the more than 90 known pneumococcal serotypes. A 10-valent pneumococcal non-typeable *Haemophilus influenzae* protein D conjugate vaccine (PHiD-CV) extended the coverage to serotypes 1, 5, and 7F, which cause a significant proportion of IPD in the developing world. PHiD-CV was licensed for use in children <2 years of age in 2009, following studies that demonstrated an immunogenicity profile comparable to that of PCV7 and acceptable safety and immunogenicity when co-administered with other paediatric vaccinations [7]–[9] and it has since been demonstrated to protect against IPD [10].

Catch-up vaccination in addition to routine vaccination of infants has been found to be an effective strategy to more rapidly achieve the population-level benefits of vaccination and extend protection to older children, who bear a substantial part of the burden of vaccine-preventable childhood diseases. The Kenya Ministry of Public Health and Sanitation introduced PHiD-CV into the routine infant vaccination programme in 2011, with a catch-up campaign in two districts to provide PHiD-CV to children 12–59 months of age. Because PHiD-CV was not licensed for use in children older than 23 months of age at that time, we aimed to assess the immunogenicity, impact on carriage, and reactogenicity of primary vaccination with PHiD-CV in children aged 12–59 months prior to the national introduction of the vaccine. We specifically aimed to evaluate whether one or two doses were required for a robust immune response and to reduce carriage. We also assessed whether the timing of the second dose had an impact on the response.

## Materials and Methods

### Population and Study Design

This phase III double-blind randomized controlled trial involving children aged 12–59 months was conducted between January–September 2010 in Malindi District, Kenya (NCT01028326). The protocol was approved by the Oxford Tropical Ethical Review Committee (No. 54-09) and the Kenya National Ethical Review Committee (SSC1635). The protocol for this trial and the supporting CONSORT checklist are available as supporting information (Protocol S1 and Checklist S1). Children were recruited at one of two health dispensaries in a rural area of Malindi District on the Kenyan coast. Humidity is high and there are two annual rainy seasons, April through July and November through December. The area is endemic for malaria, with declining transmission over the last 10 years. *H. influenzae* type b vaccine, given with diphtheria and tetanus toxoids and pertussis vaccine to infants after 6, 10 and 14 weeks, was introduced in 2001. Written informed consent was obtained from the parent or guardian of each subject prior to participation. Children were considered eligible if they did not have a serious medical condition (e.g., severe malnutrition, HIV infection, malignancy) and had not received pneumococcal conjugate vaccine or hepatitis A vaccine (HAV) from birth, or any dose of vaccine against diphtheria, tetanus, or pertussis after the first birthday.

### Vaccine and Schedules

**Figure 1:**
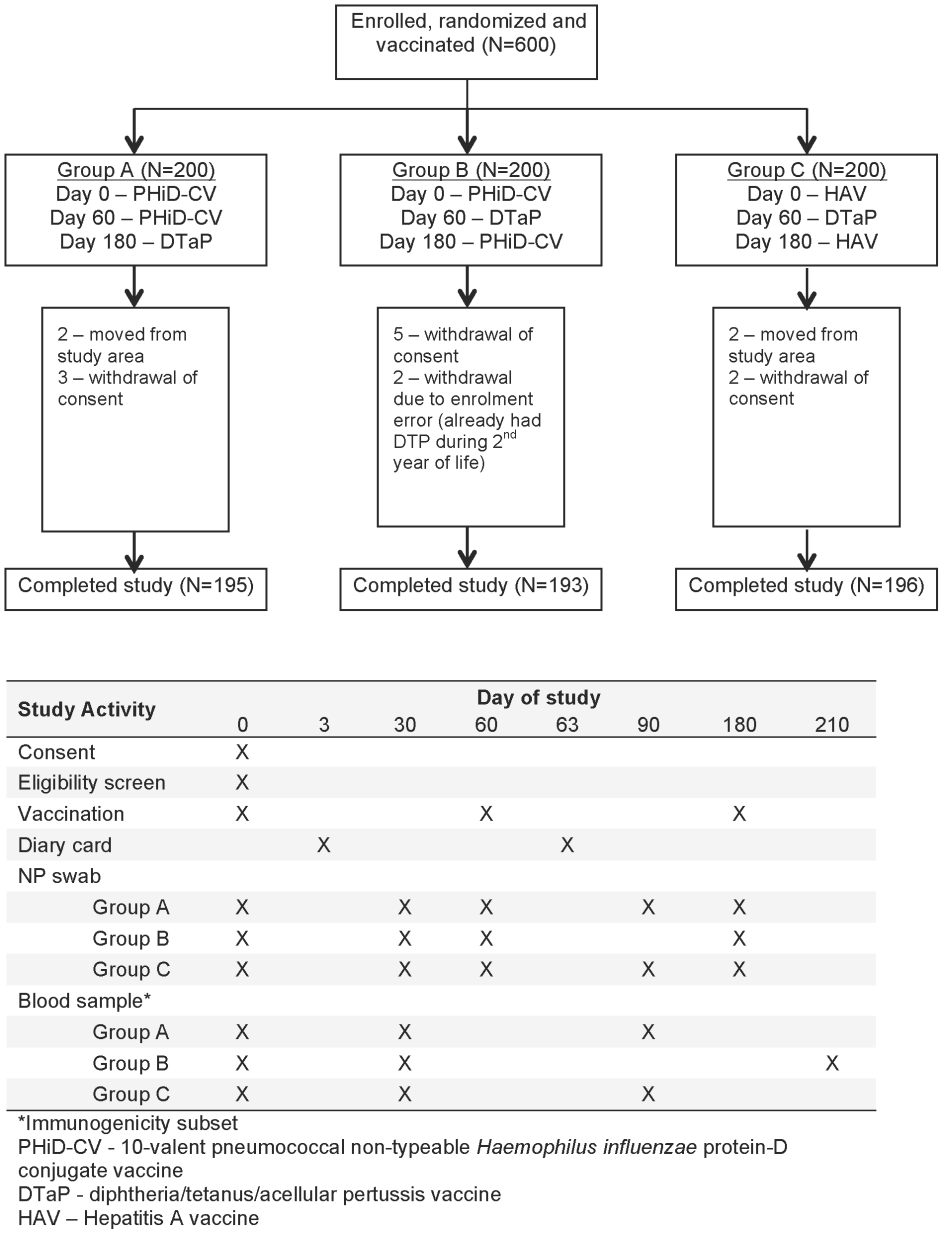
Trial profile.

Eligible children were assigned into the appropriate age stratum (12–23 months, 24–35 months, 36–47 months or 48–59 months) and one of three vaccination regimens using a computer-generated block randomization scheme that was generated by the study sponsor before the start of the trial (block size = 6). The study nurse assigned children sequentially to the next available study number. Vaccines were administered in the left deltoid muscle on days 0, 60 and 180 (Figure 1) and the sequence was PHiD-CV, PHiD-CV, diphtheria/tetanus/acellular pertussis vaccine (DTaP) in Group A; PHiD-CV, DTaP, PHiD-CV in Group B; hepatitis A vaccine (HAV), DTaP, HAV in Group C (i.e., the control group). Vaccines were provided by GlaxoSmithKline Vaccines in pre-filled syringes labelled with the randomization number and dose number; syringes were indistinguishable. The study group assignment was unblinded by the study nurse on day 180 and one dose of PHiD-CV was administered to all subjects in the control group.

### Immunogenicity

In a subset of 375 children (all subjects aged 12–23 months and the first 25 subjects enrolled into each study group for each of the older 3 age strata), blood was collected prior to vaccination, 30 days after the first vaccine visit, and either 30 days after the second vaccine visit (on day 90; Groups A, C) or 30 days after the third vaccine visit (day 210; Group B). Plasma samples were stored at −20°C or colder until analyzed.

Serotype-specific anti-capsular IgG concentrations to each of the 10 vaccine serotypes were measured by 22F-inhibition ELISA, i.e following adsorption with cell wall polysaccharide and 22F polysaccharide, in the WHO Pneumococcal Serology Reference Laboratory at the Institute of Child Health, University College, London [11].

The functional capacity of antibody to each of the 10 vaccine serotypes was measured by opsonophagocytic activity (OPA) using a modification of the HL-60 cell WHO reference method in the SGS laboratory, Belgium [12], [13]. Viable colony counts were performed after the plates were incubated overnight at 37°C in 5% CO_2_ atmosphere. OPA titers are expressed as the reciprocal of the serum dilution able to sustain ≥50% bacterial killing.

### Nasopharyngeal Carriage

Nasopharyngeal (NP) rayon swabs (Medical Wire Co, UK) were collected from all subjects at enrolment, days 30, 60, 90 (Groups A and C only) and day 180. Specimens were collected by passing the swab through the nostril, along the floor of the nasal cavity until it touched the posterior nasopharyngeal wall, where it was left for 2–3 seconds, rotated, and removed. Swabs were placed in skim-milk tryptone glucose glycerol (STGG) media and processed at the KEMRI- Wellcome Trust Programme laboratory in Kilifi, Kenya, in accordance with WHO recommendations [14]. Isolates of *Streptococcus pneumoniae* and *H. influenzae* were identified by standard microbiological methods. Pneumococci were identified from gentamicin-blood agar by optochin susceptibility testing; serotyping was performed by latex agglutination and the Quellung reaction. Typing of *H. influenzae* was performed by multiplex PCR using an IgA target that discriminates between *H. influenzae* and *Haemophilus haemolyticus* [15] and a *bex*A target to determine capsular type [16].

### Reactogenicity

Study personnel assessed all subjects 30 minutes after every dose and three days after doses 1 and 2 and documented observed local and systemic adverse events. In addition, study personnel interviewed parents/guardians to determine the subject’s peak local and systemic adverse events in the three days following doses 1 and 2. For efficiency of design, reactogenicity was not assessed three days after dose 3. Serious adverse events were recorded for all subjects throughout the study.

### Statistical Analysis

The primary outcomes were carriage and immunogenicity after two doses of PHiD-CV. The sample size was determined for these outcomes. Given an estimated background prevalence of nasopharyngeal carriage of the PHiD-CV serotypes of 25% in children in coastal Kenya, a sample size of 200 in each group would give 90% power (alpha = 0.05) to detect a difference in the vaccine-type (i.e., serotypes 1, 4, 5, 6B, 7F, 9V, 14, 18C, 19F, and 23F) carriage prevalence of 12% in recipients of 1 or 2 doses of PHiD-CV and 25% in controls, allowing for 10% attrition. With a sample size of 100 children who received PHiD-CV at study day 0 and day 60 or 180, the 95% confidence interval for finding that 95% achieved the protective antibody threshold would be 89%–98%, allowing for 10% attrition. The sample size was therefore set at 600 children (50 children in each of four age groups in each of three vaccine regimens), with a subset of 375 children followed for immunological evaluation of blood (50 children aged 12–23 months in each of the three vaccine regimens and 25 children in each of the three vaccine regimens for the older age groups). Given this sample size, the study had 88% power to detect a difference in the proportion of children that developed a seroprotective antibody concentration following one dose of vaccine of 90%, compared to 99% in those receiving a second dose of vaccine.

Anti-pneumococcal IgG concentrations and OPA titers were log-transformed for analysis. Differences between Groups A and B were tested and, if appropriate, data from these two groups were combined for analysis. Immunogenicity following one dose versus two doses of PHiD-CV was compared by a paired t-test of log values. NP carriage prevalence was compared between groups using the Chi-square test. The vaccine efficacy against carriage and its 95% confidence interval was calculated as 1 minus the age-adjusted odds ratio of carriage in PHiD-CV recipients compared to control vaccine recipients. Solicited adverse experiences were compared between groups using the Chi-square test or Fisher’s exact test. Results were interpreted using two-sided p-values for pre-specified hypotheses, with a p-value <0.05 interpreted as significant. No adjustment was made for multiple comparisons. All analyses were performed on the total vaccinated cohort. Statistical analysis was performed using STATA 11.

## Results

### Study Population

There were 600 Kenyan children (200 in each vaccination regimen) recruited during January–February 2010, of whom 584 completed the study (10 withdrew consent, four moved from the study area, and two were withdrawn because of an enrolment error; Figure 1). Subject characteristics were similar for the three study groups with males comprising 48–51% of each group and an even distribution of children in each age stratum across study groups (24–28% per stratum per group) (Table 1). Attendance during the study was high with over 97% of subjects attending the scheduled visits.

**Table 1:** Participant Characteristics.

Characteristic	All groups	Group A	Group B	Group C
	n (%)	n (%)	n (%)	n (%)
Male	297 (49.5)	99 (49.5)	102 (51)	96 (48)
MUAC (median, IQR)	15 (14.3–15.8)	15 (14.3–15.8)	15 (14.2–15.9)	15 (14.3–15.8)
Had three doses of Pentavalent vaccine	585 (97.5)	193 (96.5)	195 (97.5)	197 (98.5)
Had at least one dose of Measles vaccine	572 (95.3)	186 (93.0)	192 (96.0)	194 (97.0)
Age group 12–23 months	142 (23.7)	47 (23.5)	47 (23.5)	48 (24.0)
Age group 24–35 months	153 (25.5)	50 (25.0)	55 (27.5)	48 (24.0)
Age group 36–47 months	151 (25.2)	50 (25.0)	50 (25.0)	51 (25.5)
Age group 48–59 months	154 (25.7)	53 (26.5)	48 (24.0)	53 (26.5)

MUAC = mid upper arm circumference.

IQR = interquartile range.

### Immunogenicity Subset

375 children were enrolled in the immunogenicity cohort. Median day of follow-up blood specimen collection was as follows: day 30 (range 25–35), day 88 (range 84–97; Groups A and C); day 210 (range 198–216; Group B).

**Figure 2:**
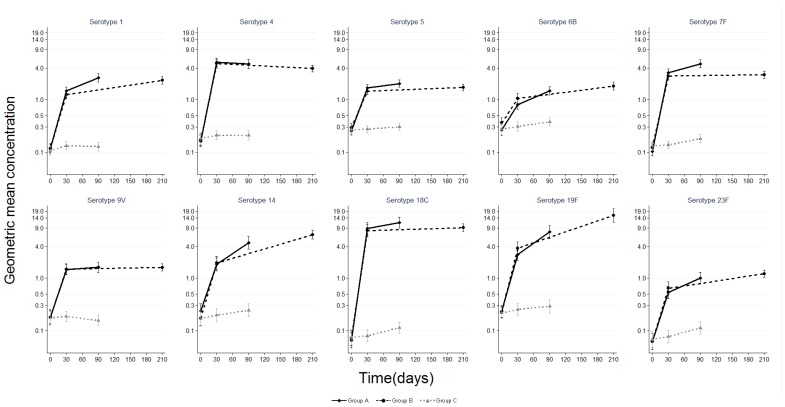
Pneumococcal serotype-specific IgG geometric mean concentration before and after PHiD-CV or control vaccine. Pneumococcal serotype-specific IgG geometric mean concentration with 95% confidence interval among children aged 12–59 months before and after PHiD-CV (Groups A and B) or control vaccine (Group C). Group A N (range) by day for GMC: day 0 (125–127); day 30 (124); day 90 (120–122) Group B N (range) by day for GMC: day 0 (121–124); day 30 (119–120); day 210 (101–115) Group C N (range) by day for GMC: day 0 (121–124); day 30 (119–121); day 90 (117–119).

**Figure 3:**
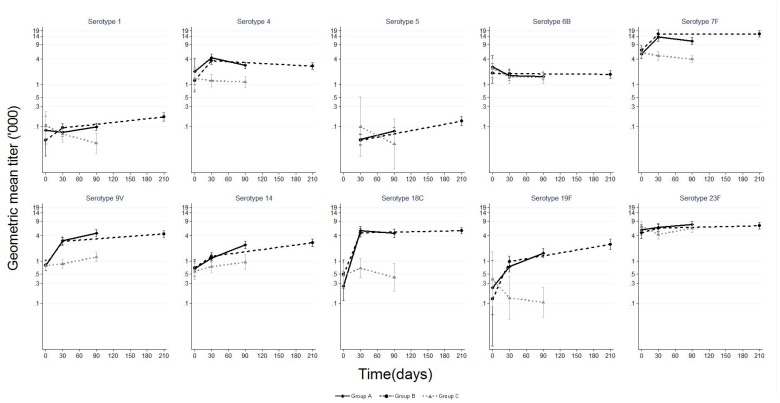
Pneumococcal serotype-specific opsonophagocytic activity geometric mean titer before and after PHiD-CV or control vaccine. Pneumococcal serotype-specific opsonophagocytic activity geometric mean titer and 95% confidence interval among children aged 12–59 months before and after PHiD-CV (Groups A and B) or control vaccine (Group C). Group A N (range) by day: day 0 (0–110); day 30 (66–124); day 90 (104–121) Group B N (range) by day: day 0 (0–102); day 30 (60–119); day 210 (97–115) Group C N (range) by day: day 0 (3–109); day 30 (2–118); day 90 (2–118) Results for serotype 5 at day 0 not available because of insufficient specimen volume.

Baseline serotype-specific anti-capsular IgG geometric mean concentrations (GMCs) and OPA geometric mean titers (GMTs) were similar for the three study groups (Figures 2 and 3). Following one dose of PHiD-CV, the IgG GMCs for all vaccine serotypes and OPA GMTs for five serotypes were significantly higher in PHiD-CV recipients compared to recipients of control vaccine. In paired analysis, a significant increase in IgG GMC was observed following a second dose of PHiD-CV for serotypes 1, 5, 6B, 14, 19F and 23F; an increase for serotypes 7F, 9V and 18C was also observed but only in Group A subjects (Table 2). The post-dose 2 GMCs were similar between Group A (PHiD-CV given two months after dose 1) and Group B (PHiD-CV given six months after dose 1) for all serotypes except for significantly higher GMCs for serotype 7F in Group A and for serotype 19F in Group B (Figure 2). In paired analysis, there was a significant increase in OPA GMTs following a second dose for serotypes 1, 5, 9V, 14, and 19F; an increase for serotype 23F was also observed but only in Group B subjects (Table 2). Of note, the OPA GMTs in recipients of two doses of PHiD-CV were not significantly different than in controls for serotypes 5, 6B and 23F (Figure 3).

**Table 2:** Ratio of serotype-specific IgG geometric mean concentration (GMC) and OPA geometric mean titer (GMT) post-dose 2 (day 90 for Group A or day 210 for Group B) : post-dose 1 (day 30).

Sero-type	IgG GMC								OPA GMT							
	Group A				Group B				Group A				Group B			
	Dose2:Dose1Ratio	LL	UL	P-value*	Dose2:Dose1Ratio	LL	UL	P-value*	Dose2:Dose1Ratio	LL	UL	P-value*	Dose2:Dose1Ratio	LL	UL	P-value*
1	1.8	1.6	2.1	<0.001	1.9	1.6	2.3	<0.001	1.8	1.4	2.3	0.001	2.1	1.5	2.9	<0.001
4	0.9	0.8	1.1	0.21	0.8	0.7	0.9	0.001	0.7	0.6	0.8	<0.001	0.8	0.7	1.0	0.02
5	1.2	1.1	1.4	0.003	1.2	1.1	1.4	0.007	2.1	1.5	2.9	<0.001	4.9	3.2	7.5	<0.001
6B	1.8	1.5	2.2	<0.001	1.7	1.4	2.2	<0.001	1.2	0.9	1.4	0.14	1.1	0.9	1.9	0.32
7F	1.5	1.3	1.6	<0.001	1.1	0.9	1.2	0.43	0.8	0.7	1.0	0.03	1.0	0.8	1.2	0.81
9V	1.2	1.0	1.3	0.01	1.0	0.9	1.2	0.65	1.5	1.2	1.7	<0.001	1.4	1.2	1.8	0.001
14	2.6	2.1	3.3	<0.001	3.1	2.4	4.0	<0.001	2.9	2.3	3.7	<0.001	2.5	1.9	3.2	<0.001
18C	1.3	1.2	1.5	<0.001	1.1	0.8	1.5	0.44	0.9	0.8	1.1	0.14	1.1	0.9	1.3	0.61
19F	2.8	2.3	3.4	<0.001	4.0	3.2	5.0	<0.001	2.8	2.2	3.7	<0.001	3.4	2.6	4.5	<0.001
23F	1.8	1.5	2.1	<0.001	1.9	1.6	2.4	<0.001	1.2	1.0	1.4	0.09	1.2	1.0	1.5	0.04

IgG GMC N (range): Group A (119–121); Group B (100–115).

OPA GMT N (range): Group A (62–120); Group B (54–113).

LL = lower limit of 95% confidence interval; UL = upper limit of 95% confidence interval.

* Paired t-test of log concentration.

**Figure 4:**
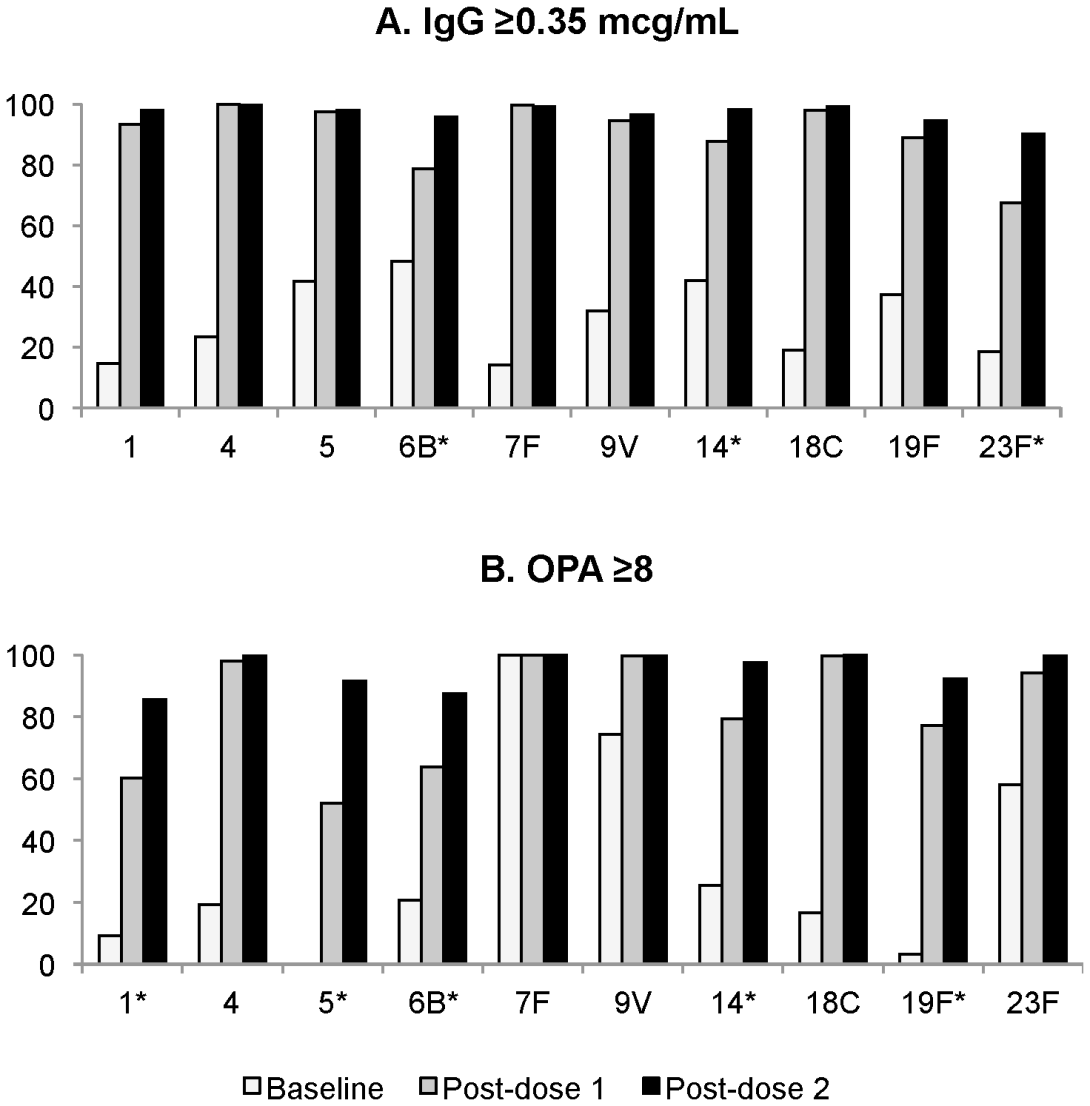
Proportion of participants with serotype-specific IgG ≥0.35 mcg/mL (A) and OPA≥8 (B). Proportion of children aged 12–59 months with serotype-specific IgG ≥0.35 mcg/mL (A) and OPA≥8 (B) at baseline and following one and two doses of PHiD-CV. Results for Groups A and B were similar so combined results are presented. Numbers of subjects sampled are shown in Figure 2 and Figure 3. See also Tables S1 and S2. *Serotypes for which the confidence intervals are non-overlapping for proportions meeting the specified threshold post-dose 1 and post-dose 2.

At day 30, the proportion of subjects with serotype-specific IgG≥0.35 mcg/mL and OPA≥8 was significantly higher in PHiD-CV recipients compared to controls for all serotypes except for OPA for serotype 7F, for which 100% of participants had OPA≥8 at all timepoints (Tables S1 and S2). Following one dose of PHiD-CV, >90% of recipients (Group A and Group B) developed IgG≥0.35 µg/mL to serotypes 1, 4, 5, 7F, 9V and 18C and OPA≥8 to serotypes 4, 7F, 9V, 18C, and 23F (Figure 4). After a second dose, >90% of recipients (Group A and Group B) had IgG≥0.35 µg/mL to all vaccine serotypes and OPA≥8 to all vaccine serotypes except 1 and 6B. The proportions of subjects above the pre-specified thresholds were similar regardless of whether the second dose was given two or six months after the first dose.

In exploratory analysis, the antibody GMCs and/or proportion of subjects with IgG≥0.35 mcg/mL after one dose of PHiD-CV were statistically significantly lower in children aged 12–23 months, compared to children aged 24–59 months for serotypes 1, 6B, 14, 19F, and 23F. A significant difference between age groups was not observed after a second dose except for lower antibody GMCs for serotype 14 in the younger age group (Table S3).

### Nasopharyngeal Carriage

**Figure 5:**
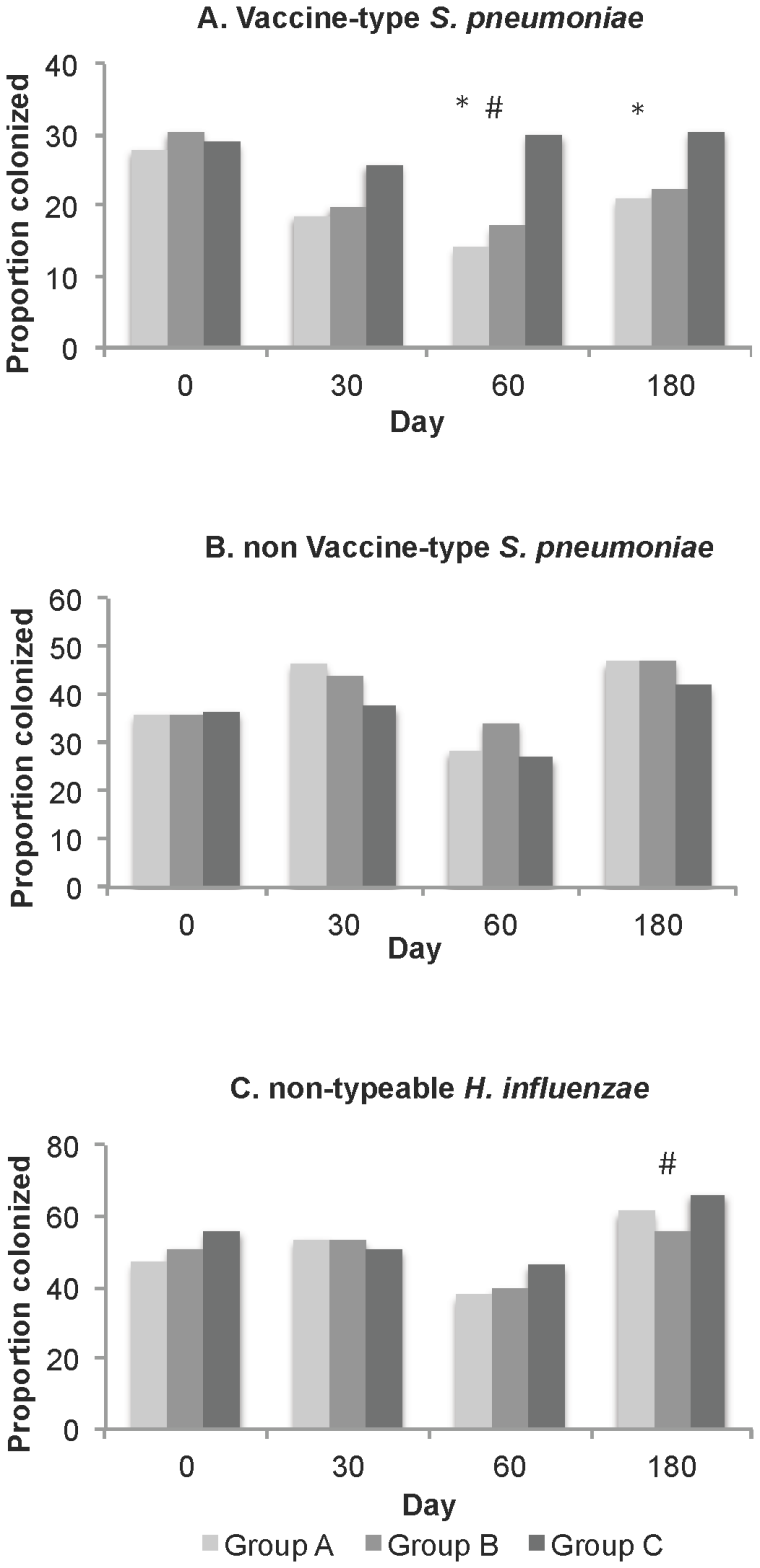
Nasopharyngeal carriage among children aged 12–59 months before and after PHiD-CV or control vaccine. Nasopharyngeal carriage of vaccine-type pneumococci (A), non-vaccine type pneumococci (B) and non-typeable *H. influenzae* (C) among children aged 12–59 months before and after PHiD-CV or control vaccine. N = 192–200 subjects per group per timepoint. See also Table S4. *p-value <0.05, comparing carriage between Group A and Group C. ^#^p-value <0.05, comparing carriage between Group B and Group C.

No major differences in the nasopharyngeal carriage prevalence of *S. pneumoniae* were observed between groups at day 0 (Figure 5; Table S4). At day 60, vaccine-type pneumococcal carriage prevalence was lower among PHiD-CV recipients compared to control subjects (Groups A/B 16% vs Group C 30%, p-value<0.001). Vaccine-type pneumococcal carriage was also lower at day 180 among PHiD-CV recipients compared to controls (Group A 21% vs Group C 31%, p-value = 0.04; Group B 22% vs Group C 31%, p-value = 0.07). This translates to a vaccine efficacy against vaccine-type carriage of 40% (94% CI: 4, 62) for Group A (ie subjects who had received two doses of PHiD-CV), and 36% (95% CI: −1, 60) for Group B (ie subjects who had received one dose of PHiD-CV). The carriage prevalence of non-vaccine type pneumococci was not significantly different in PHiD-CV recipients compared to controls at any timepoint.

No statistically significant differences in the carriage prevalence of non-typeable *H. influenzae* (NTHi) were observed between groups with the exception of lower carriage among Group B PHiD-CV recipients compared to controls at day 180 (Group B 56% vs Group C 66%; p = 0.04) (Figure 5; Table S4).

In exploratory analysis, the effect of nasopharyngeal carriage on the IgG and OPA response at day 30, 90 and 210 was analyzed for the four serotypes most commonly carried at day 0 among subjects in Groups A and B (i.e., serotypes 6B, 14, 19F, and 23F). There was no significant difference in the IgG GMCs and OPA GMTs of carriers and non-carriers with the exception of serotype 19F for which lower GMCs and GMTs were observed among carriers at day 30, day 90, and 210 and for serotype 6B, for which lower GMCs were observed at day 210 (data not shown).

### Reactogenicity

Information on local and systemic adverse experiences in the three days following vaccination was available for 600 subjects following dose 1 and for 587 subjects following dose 2. Fever was reported after dose 1 by 41% of PHiD-CV recipients compared to 26% children who received hepatitis A vaccine. Other local and systemic adverse experiences following vaccination were in the same range for the various groups after the first and second doses of vaccine (Table 3).

**Table 3:** Solicited adverse events among children aged 12–59 months during the three days following dose 1 or dose 2 of PHiD-CV or control vaccine.

Post-dose 1	Group A: PHiD CV				Group B: PHiD-CV				Group C: HAV				Group AB combined				p-value*
Sign/Symptom	n	%	LL	UL	n	%	LL	UL	n	%	LL	UL	n	%	LL	UL	AB vs C
Any swelling	97	48.5	41.4	55.7	101	50.5	43.4	57.6	87	43.5	36.5	50.7	198	49.5	44.5	54.5	0.17
Any pain	166	83.0	77.1	87.9	172	86.0	80.4	90.5	157	78.5	72.2	84.0	338	84.5	80.6	87.9	0.07
Any redness	19	9.5	5.8	14.4	25	12.5	8.3	17.9	16	8.0	4.6	12.7	44	11.0	8.1	14.5	0.25
Any irritability	130	65.0	58.0	71.6	134	67.0	60.0	73.5	131	65.5	58.5	72.1	264	66.0	61.1	70.6	0.90
Any drowsiness	15	7.5	4.3	12.1	15	7.5	4.3	12.1	12	6.0	3.1	10.2	30	7.5	5.1	10.5	0.50
Any fever	80	40.0	33.2	47.1	83	41.5	34.6	48.7	52	26.0	20.1	32.7	163	40.8	35.9	45.7	<0.001
Any local reaction	171	85.5	79.8	90.1	176	88.0	82.7	92.2	166	83.0	77.1	87.9	347	86.8	83.0	89.9	0.22
Any systemic reaction	159	79.5	73.2	84.9	163	81.5	75.4	86.6	145	72.5	65.8	78.6	322	80.5	76.3	84.3	0.03
**Post-dose 2**	**Group A: PHiD-CV**				**Group B: DTaP**				**Group C: DTaP**				**Group BC combined**				**p-value** *
**Sign/Symptom**	**n**	**%**	**LL**	**UL**	**n**	**%**	**LL**	**UL**	**n**	**%**	**LL**	**UL**	**n**	**%**	**LL**	**UL**	**A vs BC**
Any swelling	35	17.8	12.7	23.8	37	19.1	13.8	25.3	33	16.8	11.9	22.8	70	17.9	14.3	22.1	0.96
Any pain	80	40.6	33.7	47.8	65	33.5	26.9	40.6	75	38.3	31.4	45.5	140	35.9	31.1	40.9	0.27
Any redness	1	0.5	0	2.8	0	0.0	0.0	1.9	2	1.0	0.1	3.6	2	0.5	0.1	1.8	1.00
Any irritability	26	13.2	8.8	18.7	22	11.3	7.2	16.7	16	8.2	4.7	12.9	38	9.7	7.0	13.1	0.21
Any drowsiness	2	1.0	0.1	3.6	2	1.0	0.1	3.7	3	1.5	0.3	4.4	5	1.3	0.4	3.0	1.00
Any fever	53	26.9	20.8	33.7	55	28.4	22.1	35.2	60	30.6	24.2	37.6	115	29.5	25.0	34.3	0.51
Any local reaction	95	48.2	41.1	55.4	86	44.3	37.2	51.6	86	43.9	36.8	51.1	172	44.1	39.1	49.2	0.34
Any systemic reaction	72	36.5	29.8	43.7	73	37.6	30.8	44.9	75	38.3	31.4	45.5	148	37.9	33.1	43.0	0.74

LL = lower limit of 95% confidence interval; UL = upper limit of 95% confidence interval.

* Chi-square p-value or, if cell size <5, Fisher’s exact p-value.

There were no reports of grade 3 local adverse experiences (swelling >30 mm, redness >30 mm, pain that prevented use of the arm). Grade 3 drowsiness (drowsiness significant enough to prevent normal activities) was reported in one child during the 3 days following the first vaccination with PHiD-CV. A temperature of 39.0–39.4°C was documented following hepatitis A vaccine on one occasion and DTaP on one occasion; no child had a temperature ≥39.5°C.

A total of seven serious adverse events (SAEs) were reported in six subjects. SAEs were documented in five (1.3%) of 400 children who received PHiD-CV and one (0.5%) of 200 children in the control group. One subject died after being struck by a car approximately 4 hours after receiving the final study vaccination (PHiD-CV). All SAEs were considered to be unrelated to vaccination.

## Discussion

Countries aiming to include pneumococcal vaccination in the routine infant immunization schedule must consider whether or not to conduct a catch-up campaign to extend protection to older children and accelerate the population-level indirect benefits of vaccine use. In this study, we found that administration of PHiD-CV to children aged 12–59 months was immunogenic, reduced nasopharyngeal carriage of vaccine-type pneumococci and was well-tolerated. The immunogenicity of catch-up vaccination with PHiD-CV has been documented in several studies[17]–[20]; however, this is the first study to demonstrate reductions in carriage following catch-up vaccination in a developing world setting and to compare the immunogenicity of one versus two doses of vaccine.

Nasopharyngeal carriage of pneumococcus has been shown to be a key event in the pathogenesis of IPD [21], [22] and reductions in carriage among vaccinated children have been associated with reductions in carriage and IPD at the population level [23]. Among children aged 12–59 months, we found that nasopharyngeal carriage of vaccine-type pneumococci began to drop within one month of the first dose of PHiD-CV and that the reductions were statistically significant just two months following vaccination. This effect was sustained through 6 months post-vaccination, although the reduction was only statistically significant in children who had received two doses of PHiD-CV. Dagan et al also assessed the impact of catch-up dosing on carriage of vaccine-type pneumococci, albeit with a different study design and different vaccine formulation, and found similar results among toddlers in Israel followed for 24 months after vaccination with one or two doses of a nine-valent PCV or control vaccine (odds ratio for vaccine-type carriage was 0.50 [0.38–0.66]) [24]. Consistent with other trials, we did not observe an obvious impact of vaccination on carriage of NTHi [25]–[27]. In contrast, carriage surveys conducted in Kilifi, Kenya two years before and two years after introduction of PHiD-CV into the routine childhood vaccination schedule, accompanied by a catch-up campaign, revealed significant reductions in NTHi carriage [28]. Widespread, programmatic use of a vaccine might result in a greater impact on carriage than is observed in individually-randomized clinical trials because mass vaccination changes not only the susceptibility of the vaccinated individual to carriage, but also the exposure of all individuals in the community through indirect effects.

The immune response (IgG GMC, OPA GMT and proportion above pre-specified thresholds) was generally higher following the second versus the first dose of PHiD-CV, although this varied by serotype. A threshold of serotype-specific IgG ≥0.35 mcg/mL following primary vaccination has been established as the non-inferiority threshold for comparison and licensure of new PCV products [29], [30]. It has been suggested that OPA ≥8 can better predict protection against IPD [31]. The clinical relevance of these thresholds is unclear in older children receiving a catch-up schedule. A second dose of PHiD-CV was required to bring the proportion of subjects with serotype-specific IgG ≥0.35 mcg/mL above 90% for serotypes 6B, 14 and 23F and with serotype-specific OPA ≥8 above 80% for serotypes 1, 5, 6B, 14 and 19F. An increase in antibody GMC did not always correspond with an increase in OPA GMT; however, our ability to interpret these findings is limited by low sample size for some serotypes for the OPA assay. Additional work is needed to understand the relationship between these two measures and the association between OPA and protection from IPD. Responses were generally similar regardless of whether the second dose of PHiD-CV was given 2 or 6 months after the first dose. Importantly, children in the youngest age group had significantly lower antibody responses compared to older children following a single dose of PHiD-CV for several serotypes. A second dose proved particularly beneficial in younger children and for generation of functional antibody to several serotypes that are important causes of IPD locally.

As in other studies of catch-up vaccination with PHiD-CV, we found that the immune responses to 6B and 23F were less robust compared to other vaccine serotypes [17], [18], [20]. Although the serotype-specific antibody concentrations in this study were lower than observed following 1 or 2 doses of 9-valent PCV in UK toddlers, the proportions achieving the threshold of ≥0.35 mcg/mL for each serotype were comparable [32]. Antibody GMCs following one dose of vaccine in 12–23 month olds in our study appear similar to those observed in American Indian children vaccinated with 7-valent PCV for serotypes 6B, 9V, and 14; however, GMCs were higher in our study for serotypes 4, 18C and 19F and lower for serotype 23F [33].

There are several limitations to our study. We did not collect a blood specimen immediately prior to dose 2 and therefore can only comment on the post-dose 2 responses in relation to the post-dose 1 antibody GMC and OPA GMT values. Group B subjects were approximately four months older than Group A subjects when the post-dose 2 blood sample was collected; this may have obscured our ability to detect a significant difference between the two dosing schedules. PCR may not differentiate perfectly between non-typeable *H. influenzae* and *H. haemolyticus*; misclassification of *H. haemolyticus* as non-typeable *H. influenzae* could have resulted in an underestimation of vaccine effectiveness against carriage of non-typeable *H. influenzae*. In coastal Kenya, nasopharyngeal carriage of pneumococci is highest following the rainy season and lowest in the dry season [34]. The timing of our study was such that enrolment was conducted during the dry season and the day 180 NP swabs were collected just after the rainy season. However, adjustment for season did not significantly change the outcomes.

The data from this study indicate that two doses of PHiD-CV administered two to six months apart to children aged 12–59 months are well-tolerated, elicit immune responses including functional antibodies and reduce nasopharyngeal carriage of vaccine-type pneumococci. On balance, results suggest greater impact on immune responses for two doses versus one dose; however, countries considering “catch-up” immunization would need to consider the local epidemiology to determine whether the theoretical added value of a second dose justified the added cost in terms of vaccine supply and delivery. Overall, these findings suggest that countries choosing to conduct catch-up vaccination with PHiD-CV could expect the vaccine to reduce disease in the vaccinated cohort and contribute to indirect protection in unvaccinated children and adults.

## Supporting Information

Table S1 **Percentage of children with serotype-specific antibody concentration ≥0.35 mcg/ml and 95% confidence interval (CI) among children aged 12–59 months before and after vaccination with PHiD-CV (Groups A and B) or control vaccine (Group C).**
(DOCX)

Table S2 **Percentage of children with serotype-specific antibody OPA titer ≥8 and 95% confidence interval (CI) among children aged 12–59 months before and after vaccination with PHiD-CV (Groups A and B) or control vaccine (Group C).**
(DOCX)

Table S3 **Serotype specific antibody response (proportion ≥0.35 mcg/mL and geometric mean concentration [GMC]) post-dose 1 (day 30) and post-dose 2 (day 90/210) by age category (Groups A and B combined).**
(DOCX)

Table S4 **Nasopharyngeal carriage among children aged 12–59 months before and after vaccination with PHiD-CV (Groups A and B) or control vaccine (Group C).**
(DOCX)

Checklist S1 **CONSORT checklist.**
(DOC)

Protocol S1 **Trial Protocol.**
(PDF)
