# Supplementary material for: Immunogenicity, Impact on Carriage and Reactogenicity of 10-Valent Pneumococcal Non-Typeable Haemophilus influenzae Protein D Conjugate Vaccine in Kenyan Children Aged 1–4 Years: A Randomized Controlled Trial
Source: PLoS One. 2014 Jan 21;9(1):e85459. doi: 10.1371/journal.pone.0085459 (PMC3897448; doi:10.1371/journal.pone.0085459)
Supplement: Table S1 — Percentage of children with serotype-specific antibody concentration ≥0.35 mcg/ml and 95% confidence interval (CI) among children aged 12–59 months before and after vaccination with PHiD-CV (Groups A and B) or control vaccine (Group C). (DOCX) [file pone.0085459.s001.docx]

**Supplemental Table 1.** Percentage of children with serotype-specific antibody concentration ≥0.35 mcg/ml and 95% confidence interval (CI) among children aged 12-59 months before and after vaccination with PHiD-CV (Groups A and B) or control vaccine (Group C)

| **Sero-type** | **Timepoint** | **Group A** | | | | **Group B** | | | | **Group C** | | |
| --- | --- | --- | --- | --- | --- | --- | --- | --- | --- | --- | --- | --- |
|  |  | **n** | **% ≥0.35 mcg/mL** | **95% CI** | **n** | | **% ≥0.35 mcg/mL** | **95% CI** | **n** | | **% ≥0.35 mcg/mL** | **95% CI** |
| 1 | Prevaccination | 22 | 18 | 11, 25 | 15 | | 12 | 7, 19 | 15 | | 12 | 7, 19 |
|  | Post-dose 1 | 118 | 95 | 90, 98 | 110 | | 92 | 85, 96 | 20 | | 17 | 10, 24 |
|  | Post-dose 2* | 121 | 99 | 96, 100 | 110 | | 97 | 91, 99 | 20 | | 17 | 11, 25 |
| 4 | Prevaccination | 26 | 21 | 14, 29 | 33 | | 27 | 19, 35 | 35 | | 28 | 21, 37 |
|  | Post-dose 1 | 124 | 100 | 97, 100 | 120 | | 100 | 97, 100 | 38 | | 31 | 23, 41 |
|  | Post-dose 2* | 122 | 100 | 97, 100 | 114 | | 99 | 95, 100 | 37 | | 31 | 23, 40 |
| 5 | Prevaccination | 50 | 39 | 31, 48 | 55 | | 44 | 35, 54 | 50 | | 40 | 32, 50 |
|  | Post-dose 1 | 122 | 98 | 94, 100 | 116 | | 97 | 92, 99 | 52 | | 43 | 34, 52 |
|  | Post-dose 2* | 118 | 97 | 92, 99 | 113 | | 99 | 95, 100 | 53 | | 45 | 36, 55 |
| 6B | Prevaccination | 57 | 45 | 36, 54 | 64 | | 52 | 43, 61 | 57 | | 47 | 38, 56 |
|  | Post-dose 1 | 94 | 76 | 67, 83 | 98 | | 82 | 74, 88 | 63 | | 53 | 43, 62 |
|  | Post-dose 2* | 115 | 94 | 89, 98 | 111 | | 97 | 93, 100 | 61 | | 52 | 43, 62 |
| 7F | Prevaccination | 14 | 11 | 6, 18 | 22 | | 18 | 12, 26 | 21 | | 17 | 11, 25 |
|  | Post-dose 1 | 124 | 100 | 97, 100 | 119 | | 99 | 95, 100 | 23 | | 19 | 12, 27 |
|  | Post-dose 2* | 122 | 100 | 97, 100 | 113 | | 98 | 94, 100 | 26 | | 22 | 15, 30 |
| 9V | Prevaccination | 41 | 33 | 25, 42 | 39 | | 32 | 24, 41 | 42 | | 34 | 26, 43 |
|  | Post-dose 1 | 117 | 94 | 89, 98 | 113 | | 95 | 89, 98 | 42 | | 35 | 26, 45 |
|  | Post-dose 2* | 117 | 97 | 92, 99 | 110 | | 97 | 91, 99 | 37 | | 31 | 23, 40 |
| 14 | Prevaccination | 61 | 48 | 39, 57 | 43 | | 36 | 27, 45 | 43 | | 35 | 27, 44 |
|  | Post-dose 1 | 106 | 86 | 78, 91 | 107 | | 90 | 83, 95 | 42 | | 35 | 27, 45 |
|  | Post-dose 2* | 116 | 97 | 92, 99 | 101 | | 100 | 96, 100 | 42 | | 37 | 28, 46 |
| 18C | Prevaccination | 22 | 17 | 11, 25 | 26 | | 21 | 14, 29 | 24 | | 19 | 13, 27 |
|  | Post-dose 1 | 124 | 100 | 97, 100 | 115 | | 96 | 91, 99 | 25 | | 21 | 14, 29 |
|  | Post-dose 2* | 122 | 100 | 97, 100 | 113 | | 98 | 94, 100 | 30 | | 25 | 18, 34 |
| 19F | Prevaccination | 44 | 35 | 27, 44 | 48 | | 40 | 31, 49 | 48 | | 40 | 31, 49 |
|  | Post-dose 1 | 111 | 90 | 83, 94 | 106 | | 88 | 81, 94 | 52 | | 43 | 34, 52 |
|  | Post-dose 2* | 114 | 93 | 88, 97 | 110 | | 96 | 90, 99 | 50 | | 42 | 33, 52 |
| 23F | Prevaccination | 23 | 18 | 12, 26 | 23 | | 19 | 12, 27 | 21 | | 17 | 11, 25 |
|  | Post-dose 1 | 79 | 64 | 55, 72 | 85 | | 71 | 62, 79 | 22 | | 18 | 12, 26 |
|  | Post-dose 2* | 105 | 87 | 79, 92 | 108 | | 94 | 88, 98 | 30 | | 26 | 18, 35 |

*day 90 for Group A and day 210 for Group B

Group A N (range) by day: 0 (125-127); 30 (124-124); 90 (120-122)

Group B N (range) by day: 0 (121-124); 30 (119-120); 210 (101-115)

Group C N (range) by day: 0 (121-124); 30 (119-121); 90 (117-119)
