# Supplementary material for: Immunogenicity, Impact on Carriage and Reactogenicity of 10-Valent Pneumococcal Non-Typeable Haemophilus influenzae Protein D Conjugate Vaccine in Kenyan Children Aged 1–4 Years: A Randomized Controlled Trial
Source: PLoS One. 2014 Jan 21;9(1):e85459. doi: 10.1371/journal.pone.0085459 (PMC3897448; doi:10.1371/journal.pone.0085459)
Supplement: Table S2 — Percentage of children with serotype-specific antibody OPA titer ≥8 and 95% confidence interval (CI) among children aged 12–59 months before and after vaccination with PHiD-CV (Groups A and B) or control vaccine (Group C). (DOCX) [file pone.0085459.s002.docx]

**Supplemental Table 2.** Percentage of children with serotype-specific antibody OPA titer ≥8 and 95% confidence interval (CI) among children aged 12-59 months before and after vaccination with PHiD-CV (Groups A and B) or control vaccine (Group C)

| **Sero-type** | **Timepoint** | **Group A** | | | **Group B** | | | **Group C** | | |
| --- | --- | --- | --- | --- | --- | --- | --- | --- | --- | --- |
|  |  | **n** | **% ≥8** | **95% CI** | **n** | **% ≥8** | **95% CI** | **n** | **% ≥8** | **95% CI** |
| 1 | Prevaccination | 13 | 10 | 6, 17 | 10 | 8 | 4, 14 | 12 | 10 | 5, 16 |
|  | Post-dose 1 | 77 | 62 | 53, 71 | 69 | 58 | 49, 67 | 19 | 16 | 10, 23 |
|  | Post-dose 2* | 104 | 86 | 79, 92 | 98 | 85 | 77, 91 | 12 | 10 | 5, 17 |
| 4 | Prevaccination | 20 | 17 | 11, 25 | 25 | 22 | 15, 30 | 28 | 24 | 17, 33 |
|  | Post-dose 1 | 121 | 98 | 94, 100 | 116 | 98 | 93, 100 | 44 | 38 | 29, 47 |
|  | Post-dose 2* | 120 | 99 | 96, 100 | 114 | 100 | 97, 100 | 41 | 36 | 27, 46 |
| 5 | Prevaccination | 0 | 0 | 0, 3 | 0 | 0 | 0, 3 | 3 | 2 | 2, 7 |
|  | Post-dose 1 | 66 | 54 | 45, 63 | 60 | 50 | 41, 59 | 2 | 2 | 0, 6 |
|  | Post-dose 2* | 111 | 91 | 84, 95 | 105 | 92 | 86, 96 | 2 | 2 | 0, 6 |
| 6B | Prevaccination | 21 | 19 | 12, 28 | 25 | 22 | 15, 31 | 24 | 23 | 15, 32 |
|  | Post-dose 1 | 70 | 62 | 52, 71 | 73 | 66 | 56, 75 | 39 | 36 | 27, 46 |
|  | Post-dose 2* | 105 | 89 | 82, 94 | 97 | 86 | 78, 92 | 36 | 31 | 2, 41 |
| 7F | Prevaccination | 110 | 100 | 97, 100 | 102 | 100 | 96, 100 | 109 | 100 | 97, 100 |
|  | Post-dose 1 | 124 | 100 | 97, 100 | 118 | 100 | 97, 100 | 118 | 100 | 97, 100 |
|  | Post-dose 2* | 121 | 100 | 97, 100 | 115 | 100 | 97, 100 | 118 | 100 | 97, 100 |
| 9V | Prevaccination | 67 | 70 | 60, 79 | 69 | 79 | 69, 87 | 69 | 73 | 63, 82 |
|  | Post-dose 1 | 122 | 99 | 96, 100 | 119 | 100 | 97, 100 | 83 | 80 | 71, 87 |
|  | Post-dose 2* | 116 | 99 | 95, 100 | 110 | 100 | 97, 100 | 63 | 59 | 49, 68 |
| 14 | Prevaccination | 30 | 28 | 20, 38 | 25 | 23 | 15, 32 | 26 | 25 | 17, 34 |
|  | Post-dose 1 | 90 | 76 | 67, 83 | 98 | 83 | 75, 89 | 44 | 39 | 30, 48 |
|  | Post-dose 2* | 118 | 98 | 94, 100 | 106 | 96 | 91, 99 | 30 | 27 | 19, 36 |
| 18C | Prevaccination | 16 | 15 | 9, 24 | 17 | 19 | 11, 28 | 22 | 25 | 16, 35 |
|  | Post-dose 1 | 117 | 99 | 95, 100 | 113 | 100 | 97, 100 | 20 | 20 | 13, 29 |
|  | Post-dose 2* | 118 | 100 | 97, 100 | 112 | 100 | 97, 100 | 11 | 11 | 6, 18 |
| 19F | Prevaccination | 6 | 5 | 2, 10 | 2 | 2 | 0, 6 | 6 | 5 | 2, 11 |
|  | Post-dose 1 | 93 | 76 | 67, 83 | 93 | 79 | 70, 86 | 6 | 5 | 2, 10 |
|  | Post-dose 2* | 110 | 92 | 85, 96 | 104 | 93 | 86, 97 | 9 | 8 | 4, 14 |
| 23F | Prevaccination | 66 | 62 | 52, 71 | 53 | 54 | 44, 64 | 71 | 61 | 52, 70 |
|  | Post-dose 1 | 115 | 94 | 88, 97 | 112 | 95 | 89, 98 | 77 | 66 | 57, 74 |
|  | Post-dose 2* | 115 | 100 | 97, 100 | 113 | 99 | 95, 100 | 60 | 59 | 49, 69 |

*****day 90 for Group A and day 210 for Group B

Group A N (range) by day: 0 (0-110); 30 (66-124); 90(104-121)

Group B N (range) by day: 0 (0-102); 30 (60-119); 210 (97-115)

Group C N (range) by day: 0 (3-109); 30 (2-118); 90 (2-118)
