# Supplementary material for: Immunogenicity, Impact on Carriage and Reactogenicity of 10-Valent Pneumococcal Non-Typeable Haemophilus influenzae Protein D Conjugate Vaccine in Kenyan Children Aged 1–4 Years: A Randomized Controlled Trial
Source: PLoS One. 2014 Jan 21;9(1):e85459. doi: 10.1371/journal.pone.0085459 (PMC3897448; doi:10.1371/journal.pone.0085459)
Supplement: Table S3 — Serotype specific antibody response (proportion ≥0.35 mcg/mL and geometric mean concentration [GMC]) post-dose 1 (day 30) and post-dose 2 (day 90/210) by age category (Groups A and B combined). (DOCX) [file pone.0085459.s003.docx]

**Supplemental Table 3.** Serotype specific antibody response (proportion ≥0.35 mcg/mL and geometric mean concentration [GMC]) post-dose 1 (day 30) and post-dose 2 (day 90/210) by age category (Groups A and B combined)

|  |  | **Post-dose 1** | | | | | | | | | | **Post-dose 2** | | | | | | | |  |
| --- | --- | --- | --- | --- | --- | --- | --- | --- | --- | --- | --- | --- | --- | --- | --- | --- | --- | --- | --- | --- |
| **Sero-type** | **Age group (months)** | **n** | **% ≥0.35 mcg/mL** | | | | | **GMC** | | | |  | **% ≥0.35 mcg/mL** | | | **GMC** | | |  |  |
|  |  |  | **%** | | **LL** | **UL** | | **value** | **LL** | **UL** | | **n** | **%** | **LL** | **UL** | **value** | **LL** | **UL** |  |  |
| 1 | 12-23 | 82 | | 89 | 81 | | 95 | 1.06 | 0.89 | 1.26 | 89 | | 97.8 | 92.3 | 99.7 | 2.52 | 2.12 | 3.00 | | |
|  | 24-59 | 146 | | 96 | 92 | | 99 | 1.56 | 1.37 | 1.79 | 142 | | 97.9 | 94.1 | 99.6 | 2.47 | 2.15 | 2.84 | | |
| 4 | 12-23 | 92 | | 100 | 96 | | 100 | 4.82 | 4.07 | 5.70 | 92 | | 100 | 96.1 | 100 | 4.72 | 4.04 | 5.52 | | |
|  | 24-59 | 152 | | 100 | 98 | | 100 | 5.18 | 4.54 | 5.90 | 144 | | 99.3 | 96.2 | 100 | 4.19 | 3.70 | 4.74 | | |
| 5 | 12-23 | 87 | | 95 | 88 | | 98 | 1.31 | 1.11 | 1.54 | 88 | | 95.7 | 89.2 | 98.8 | 1.74 | 1.49 | 2.03 | | |
|  | 24-59 | 151 | | 99 | 96 | | 100 | 1.74 | 1.54 | 1.97 | 143 | | 99.3 | 96.2 | 100 | 1.96 | 1.73 | 2.22 | | |
| 6B | 12-23 | 55 | | 60 | 49 | | 70 | 0.49 | 0.40 | 0.61 | 87 | | 94.6 | 87.8 | 98.2 | 1.51 | 1.26 | 1.80 | | |
|  | 24-59 | 137 | | 90 | 84 | | 94 | 1.35 | 1.15 | 1.59 | 139 | | 96.5 | 92.1 | 98.9 | 1.72 | 1.49 | 1.98 | | |
| 7F | 12-23 | 92 | | 100 | 96 | | 100 | 2.76 | 2.36 | 3.24 | 92 | | 100 | 96.1 | 100 | 3.67 | 3.12 | 4.31 | | |
|  | 24-59 | 151 | | 99 | 96 | | 100 | 3.25 | 2.87 | 3.67 | 143 | | 98.6 | 95.1 | 99.8 | 3.94 | 3.47 | 4.48 | | |
| 9V | 12-23 | 84 | | 91 | 84 | | 96 | 1.23 | 1.02 | 1.48 | 86 | | 93.5 | 86.3 | 97.6 | 1.67 | 1.40 | 1.98 | | |
|  | 24-59 | 146 | | 97 | 92 | | 99 | 1.64 | 1.41 | 1.89 | 141 | | 98.6 | 95 | 99.8 | 1.56 | 1.36 | 1.80 | | |
| 14 | 12-23 | 71 | | 77 | 67 | | 85 | 0.87 | 0.66 | 1.14 | 83 | | 97.6 | 91.8 | 99.7 | 4.21 | 3.34 | 5.30 | | |
|  | 24-59 | 142 | | 94 | 89 | | 97 | 3.02 | 2.43 | 3.74 | 134 | | 98.5 | 94.8 | 99.8 | 6.58 | 5.48 | 7.89 | | |
| 18C | 12-23 | 91 | | 99 | 94 | | 100 | 7.89 | 6.15 | 10.13 | 91 | | 98.9 | 94.1 | 100 | 10.35 | 8.73 | 12.27 | | |
|  | 24-59 | 148 | | 97 | 93 | | 99 | 8.75 | 7.21 | 10.62 | 144 | | 99.3 | 96.2 | 100 | 10.17 | 8.88 | 11.65 | | |
| 19F | 12-23 | 72 | | 78 | 68 | | 86 | 1.80 | 1.32 | 2.44 | 83 | | 90.2 | 82.2 | 95.4 | 7.86 | 5.60 | 11.03 | | |
|  | 24-59 | 145 | | 95 | 91 | | 98 | 4.60 | 3.63 | 5.83 | 141 | | 97.2 | 93.1 | 99.2 | 13.26 | 10.12 | 17.38 | | |
| 23F | 12-23 | 55 | | 60 | 49 | | 70 | 0.38 | 0.30 | 0.50 | 78 | | 85.7 | 76.8 | 92.2 | 0.97 | 0.81 | 1.18 | | |
|  | 24-59 | 109 | | 72 | 64 | | 79 | 0.75 | 0.61 | 0.92 | 135 | | 93.1 | 87.7 | 96.6 | 1.18 | 1.02 | 1.37 | | |

LL = lower limit of 95% confidence interval; UL=upper limit of 95% confidence interval
