# Supplementary material for: Immunogenicity, Impact on Carriage and Reactogenicity of 10-Valent Pneumococcal Non-Typeable Haemophilus influenzae Protein D Conjugate Vaccine in Kenyan Children Aged 1–4 Years: A Randomized Controlled Trial
Source: PLoS One. 2014 Jan 21;9(1):e85459. doi: 10.1371/journal.pone.0085459 (PMC3897448; doi:10.1371/journal.pone.0085459)
Supplement: Table S4 — Nasopharyngeal carriage among children aged 12–59 months before and after vaccination with PHiD-CV (Groups A and B) or control vaccine (Group C). (DOCX) [file pone.0085459.s004.docx]

**Supplemental Table 4.** Nasopharyngeal carriage among children aged 12-59 months before and after vaccination with PHiD-CV (Groups A and B) or control vaccine (Group C)

|  | **Group A** | | | **Group B** | | | **Group C** | | | **P-value*** | | |
| --- | --- | --- | --- | --- | --- | --- | --- | --- | --- | --- | --- | --- |
|  | **n** | **%** | **95% CI** | **n** | **%** | **95% CI** | **n** | **%** | **95% CI** | **A vs C** | **B vs C** | **AB vs C** |
| ***S. pneumoniae*** | | | | | | | | | | | | |
| Day 0 | 128 | 64 | 57, 71 | 133 | 67 | 60, 73 | 131 | 66 | 59, 72 |  |  | 0.95 |
| Day 30 | 129 | 65 | 58, 72 | 125 | 64 | 57, 71 | 125 | 64 | 56, 70 |  |  | 0.81 |
| Day 60 | 84 | 43 | 36, 50 | 99 | 51 | 44, 59 | 112 | 57 | 50, 64 |  |  | 0.02 |
| Day 90† | 93 | 48 | 41, 55 | -- |  |  | 89 | 46 | 39, 53 |  |  |  |
| Day 180 | 132 | 68 | 61, 75 | 134 | 69 | 62, 76 | 142 | 72 | 66, 79 | 0.34 | 0.51 |  |
| **vaccine-type *S. pneumoniae*** | | | | | | | | | | | | |
| Day 0 | 56 | 28 | 22, 35 | 61 | 31 | 24, 37 | 58 | 29 | 23, 36 |  |  | 0.95 |
| Day 30 | 37 | 19 | 14, 25 | 39 | 20 | 15, 26 | 51 | 26 | 20, 33 |  |  | 0.07 |
| Day 60 | 28 | 14 | 10, 20 | 33 | 17 | 12, 23 | 59 | 30 | 24, 37 |  |  | <0.001 |
| Day 90† | 31 | 16 | 11, 22 | -- |  |  | 39 | 20 | 15, 26 |  |  |  |
| Day 180 | 41 | 21 | 16, 28 | 43 | 22 | 17, 29 | 60 | 31 | 24, 38 | 0.03 | 0.06 |  |
| **non-vaccine type *S. pneumoniae*** | | | | | | | | | | | | |
| Day 0 | 72 | 36 | 29, 43 | 72 | 36 | 29, 43 | 73 | 37 | 30, 44 |  |  | 0.90 |
| Day 30 | 92 | 47 | 39, 54 | 86 | 44 | 37, 51 | 74 | 38 | 31, 45 |  |  | 0.08 |
| Day 60 | 56 | 28 | 22, 35 | 66 | 34 | 28, 41 | 53 | 27 | 21, 34 |  |  | 0.29 |
| Day 90† | 62 | 32 | 25, 39 | -- |  |  | 50 | 26 | 20, 32 |  |  |  |
| Day 180 | 91 | 47 | 40, 54 | 91 | 47 | 40, 54 | 82 | 42 | 35, 49 | 0.31 | 0.29 |  |
| **non-typeable *H. influenzae*** | | | | | | | | | | | | |
| Day 0 | 94 | 47 | 40, 54 | 102 | 51 | 44, 58 | 112 | 56 | 49, 63 |  |  | 0.11 |
| Day 30 | 106 | 54 | 46, 61 | 104 | 53 | 46, 60 | 100 | 51 | 44, 58 |  |  | 0.56 |
| Day 60 | 74 | 38 | 31, 45 | 76 | 39 | 32, 47 | 91 | 46 | 39, 54 |  |  | 0.06 |
| Day 90† | 93 | 48 | 41, 55 | -- |  |  | 92 | 47 | 40, 54 |  |  |  |
| Day 180 | 119 | 61 | 54, 68 | 108 | 56 | 49, 63 | 130 | 66 | 59, 73 | 0.31 | 0.04 |  |

*Chi-square p-value

†Day 90 specimen not collected in Group B subjects
