# Supplementary material for: Immunogenicity, Impact on Carriage and Reactogenicity of 10-Valent Pneumococcal Non-Typeable Haemophilus influenzae Protein D Conjugate Vaccine in Kenyan Children Aged 1–4 Years: A Randomized Controlled Trial
Source: PLoS One. 2014 Jan 21;9(1):e85459. doi: 10.1371/journal.pone.0085459 (PMC3897448; doi:10.1371/journal.pone.0085459)
Supplement: Protocol S1 — Trial Protocol. (PDF) [file pone.0085459.s006.pdf]

**Research Protocol for the Scientific Steering Committee**
**Kenya Medical Research Institute**

## I. Title of the Project

Immunogenicity and reactogenicity of 10-valent pneumococcal conjugate vaccine (PCV10) in children aged 12-59 months

## II. Investigators and Institutional Affiliations


Principal Investigator

Laura Hammitt, KEMRI CGMR-Coast, Kilifi, Kenya

Co-Investigators

Mahfudh Bashraheil, Clinical Trials Facility, KEMRI CGMR-Coast, Kilifi, Kenya
Morris Buni, Medical Superintendent, Malindi District Hospital
Roma Chilengi, KEMRI CGMR-Coast, Kilifi, Kenya
Daniel Frank, University of Colorado, Boulder, USA
David Goldblatt, Institute of Child Health, London, UK
Fauzat Hamid, KEMRI CGMR-Coast, Malindi, Kenya
Ali Hussein, DMOH, Malindi, Kenya
Tatu Kamau, Division of Vaccines and Immunization, Ministry of Health, Nairobi, Kenya
Susan Morpeth, KEMRI CGMR-Coast, Kilifi, Kenya
Daniel Muli, KEMRI CGMR-Coast
Anthony Scott, KEMRI CGMR-Coast, Kilifi, Kenya
Benjamin Tsofa, KEMRI CGMR-Coast, Kilifi, Kenya
Britta Urban, KEMRI CGMR-Coast, Kilifi, Kenya


## III. Abstract


Pneumococcal conjugate vaccine (PCV) has been found to be highly efficacious against invasive pneumococcal disease (IPD). In Kenya, the Division of Vaccines and Immunization anticipates introducing PCV into the childhood immunization schedule in 2010. The Ministry of Health, in partnership with KEMRI CGMR-Coast, plans to assess the impact of vaccine introduction on IPD and determine the cost-effectiveness of the vaccine. In districts designated for evaluation and monitoring of the vaccine program, a catch-up campaign will commence simultaneously with introduction of PCV into the routine infant vaccination schedule. The catch-up campaign will aim to vaccinate children up to 5 years of age in order to more rapidly achieve the direct and indirect benefits of pneumococcal vaccination.

Until recently, the Ministry of Health was planning to introduce the 7-valent pneumococcal conjugate vaccine (PCV7) in Kenya. However, it is now expected that the 10-valent pneumococcal conjugate vaccine (PCV10) will be made available in Kenya through the

support of the GAVI Alliance.  PCV10, marketed by GlaxoSmithKline as Synflorix™, has been found to be safe and immunogenic in infants and young children. It is licensed for use in children <2 years in Europe, Canada and Australia. The immunogenicity of PCV10 has not been studied in older children. To be able to evaluate the impact of the catch-up campaign, it is important to understand the immune response to PCV10 in children up to age 5 years. This protocol describes a randomized, controlled trial to assess the reactogenicity and immunogenicity of 1 or 2 doses of PCV10, compared to doses of hepatitis A vaccine and DTaP.

**IV. Introduction/Background**

Invasive pneumococcal disease (IPD) is a major cause of morbidity and mortality in Kenya [1, 2]. In countries where pneumococcal conjugate vaccine (PCV) has been introduced into the childhood immunization schedule, it has reduced the incidence of IPD by up to 75% among children aged <5 years [3]. Reductions in IPD among unvaccinated children and adults have also been achieved through decreased transmission of pneumococcal infection from younger, vaccinated children [3-5].

The currently available formulations of paediatric pneumococcal vaccine contain polysaccharides from different strains of pneumococcal bacteria conjugated to a carrier protein. The 7-valent pneumococcal conjugate vaccine (PCV7) offers protection against serotypes 4, 6B, 9V, 14, 18C, 19F, 23F and has been used throughout the developed world since 2001. A 10-valent vaccine (PCV10; Synflorix™) recently developed by GlaxoSmithKline extends protection to serotypes 1, 5 and 7F.  As recommended by the WHO, the assessment of the potential efficacy of PCV10 against IPD has been based on a comparison of immune responses to the 7 serotypes shared between PCV10 and PCV7. Studies in Europe and South America have found that the immunogenicity profile of PCV10 is comparable to that of PCV7 [6] and that the vaccine is safe and immunogenic when co-administered with other common paediatric vaccinations [7-9]. Based on this information, PCV10 has been licensed for use in children < 2 years of age in Europe, Canada, and Australia.

The Kenya Ministry of Health anticipates introducing PCV10 into the routine childhood immunization schedule in 2010. PCV10 has the potential to prevent more IPD than the

currently available 7-valent vaccine by offering protection against three additional pneumococcal serotypes. The 3 additional serotypes (1, 5, and 7F) are particularly important causes of IPD in the developing world and the introduction of this vaccine is expected to result in significant reduction in paediatric morbidity and mortality. The 10 serotypes included in the new vaccine are responsible for approximately 70% of all cases of IPD in children younger than 5 years of age in Kenya. The impact of vaccine introduction will be assessed in several studies (SSC 1433). In districts designated for evaluation and monitoring of the vaccine program (including Kilifi District), a catch-up campaign will commence simultaneously with the introduction of PCV10 into the EPI schedule. In order to more rapidly attain the effects of the vaccination programme, and to offer protection to children beyond the infant period, catch-up campaign in Kilifi District and Bondo District will aim to provide 2 doses of PCV10 to all children aged >1 year and <5 years.

Individuals vaccinated with PCV generate immunoglobulin G (IgG) antibody responses to the strains of pneumococci contained in the vaccine; however, the serum antibody wanes over time [10]. A serotype-specific IgG antibody concentration of 0.35 mcg/mL has been established as the threshold level that is thought to convey protection against invasive pneumococcal disease. The ability of antibodies to kill bacteria (measured as opsonophagocytic activity) is another important marker of protection, which has also been shown to decline with time. The failure of persistence of IgG to capsular polysaccharides after immunization and the decline in functional antibodies may be overcome by the subsequent administration of a booster dose of conjugate vaccine. Among toddlers vaccinated with PCV7, there was little difference in the antibody responses among those who received either one or two doses of vaccine [11]. However, for some serotypes, antibody titres and functional activity were lower in children receiving a single dose of PCV10 at 18 months, compared to children who had received a primary series in infancy, followed by a booster dose at 18 months [6]. The need for one or two doses of vaccine in previously unvaccinated toddlers is unknown. Additionally, although PCV10 has been studied as a booster dose given during the second year of life, the immunogenicity of primary vaccination with one or two doses of PCV10 in the second year of life has not been established. In the absence of a booster dose of vaccine, long-lasting protection may persist, despite low serotype-specific antibody concentrations, if primary vaccination successfully

induces an antigen-specific memory B-cell response. Memory B-cells are long-lasting immune cells that are responsible for rapid rises in antibody following re-exposure to an antigen.  Little is known about the development of memory B cells following PCV vaccination in childhood, nor about the impact of immune system priming via nasopharyngeal carriage of pneumococci. A recent study suggests that 2 doses of PCV7 are required to generate memory B-cell frequencies equivalent to that seen in adults [12]. Understanding the IgG responses and B-cell responses to PCV10 vaccination in toddlers will help determine the most appropriate vaccination schedule for children in this age group.

In addition to generating a serum antibody response, vaccination with PCV also results in decreased nasopharyngeal (NP) carriage of vaccine-type pneumococci [13-15]. Decreased transmission of vaccine-type pneumococci from vaccinated children to unvaccinated individuals has lead to declines in PCV7-type IPD in all age classes [16]. Studies suggest that there is minimal impact on carriage one-month following primary vaccination and that a booster dose of vaccine may be more effective in reducing PCV7-type carriage, compared to a primary series or single dose of vaccine [17, 18]. The importance of one vs two doses of vaccine in the reduction of PCV-type carriage in previously unvaccinated toddlers is unknown.

In the United States, where PCV7 use is widespread, reductions in the carriage prevalence of PCV7-type pneumococci have been accompanied by increases in carriage of non-vaccine serotypes, with a resultant increase in non-PCV7 type IPD [19].  Although the increase in non-PCV7 type IPD is small compared to the overall decrease in IPD, there are some subsets of the population for which the incidence of replacement disease has increased substantially. Knowing the impact of PCV10 vaccination on NP carriage will be essential to understanding the direct and indirect (or “herd”) effects of vaccination in Kenya.

The ecology of the nasophayrnx is complex, with numerous interactions between bacterial species, but these are not well understood. Because PCV10 uses protein D from non-typeable *Haemophilus influenzae* (NTHi) as its carrier protein it also offers potential protection against infections caused by NTHi, an important cause of otitis media and respiratory tract infections [20]. Changes in pneumococcal and NTHi carriage may have

other effects, as well. Multiple studies have suggested an inverse relationship between carriage of *S pneumoniae* and *S aureus,* including two studies that identified negative correlation for co-colonization with PCV7-type pneumococci but not non-PCV7 strains. This has led to speculation that PCV use may result in a shift, not only toward non-PCV type carriage, but also toward higher *S aureus* carriage rates in children [21, 22]. Culture of NP swabs is one way to identify other colonizing bacteria. However, this is labour intensive and it may provide a limited picture because it inherently favours identification of the more easily grown species in a mixed-microbial community. Recently, culture-independent molecular methods of microbial identification and characterization have been developed and applied in the context of microbial ecology [23]. Because colonization serves as a reservoir for transmission and can precede invasive disease, it is also important to understand the impact of vaccine on bacterial colonization of the nasopharynx. The use of new molecular techniques will allow characterization of the impact of vaccine on nasopharyngeal flora.

This study will assess the immune responses and effect on NP carriage following receipt of PCV10 in children aged 12-59 months. Vaccination of children aged 24-59 months represents an "off-label" use of PCV10; however, the vaccine is licensed for use in infants and children up to 2 years of age. Children in the control group will be vaccinated with DTaP (Infanrix™) and hepatitis A vaccine (HAVRIX™). The WHO recommends a booster dose of DTaP after the first year of life but this is not currently available in Kenya as part of the routine vaccination programme.  It is anticipated that following the conclusion of the study, the Division of Vaccines and Immunization will proceed with nationwide introduction of PCV10, including a catch-up campaign for children aged 1-5 years in Kilifi District and Bondo District. Notably, because Malindi is not one of the districts targeted for a catch-up vaccine campaign, none of the study participants would be expected to receive PCV10 outside the context of the study. Because PCV10 is likely to be beneficial in this age group, study participants in the control group will given one dose of PCV10 on the final study visit, in accordance with WHO recommendations for catch-up dosing of PCV7.

## V. Justification for the Study

*Community and public health benefits*

Pneumococcal conjugate vaccine is highly efficacious against invasive pneumococcal disease. The pneumococcal vaccine formulation that will be made available in Kenya (PCV10) has not been studied in the age group of children who will be eligible for the catch-up campaign. Use of a catch-up campaign is an important way to rapidly attain the direct and indirect benefits of vaccination. In order to accurately evaluate the impact of the catch-up campaign in Kenya, and to help inform other policy makers outside of Kenya about the optimal regimen for future catch-up campaigns, it is necessary to understand the effect of one and two doses of PCV10 on serum antibodies and nasopharyngeal carriage in children aged 12-59 months.

*Individual participant benefits*

All study participants will receive the protection offered from vaccination with DTaP and any protection offered by PCV10 (not yet been established in this age group). Control group participants will also receive hepatitis A vaccine. DTaP booster vaccination is recommended by the WHO but is not part of routine childhood immunizations in Kenya. Hepatitis A vaccine is highly effective in preventing hepatitis A infection and is recommended for all children >1 year of age in the United States, but is not used in Kenya because it is not considered cost-effective [24].

**VI. Null Hypothesis**

1. Vaccination with two doses of PCV10 has no effect on the carriage prevalence of PCV10 types when compared to unvaccinated controls.
2. Vaccination with two doses of PCV10 has no effect on the serotype-specific anti-capsular IgG concentration when compared to unvaccinated controls.
3. Vaccination with one dose of PCV10 has no effect on the carriage prevalence of PCV10 types when compared to unvaccinated controls.

**VII. Objectives**

a) General Objectives

To determine the immunogenicity and reactogenicity of 1 or 2 doses of PCV10 in children aged 12-59 months and to determine the effect of vaccination on the carriage prevalence of PCV10 types.

b) Specific Objectives

1. To estimate the absolute reduction in the prevalence of vaccine-serotype nasopharyngeal carriage attributable to immunisation and evaluate the effect of immunisation on the prevalence of carriage of non-vaccine type pneumococci, *Haemophilus influenzae, Staphylococus aureus*, coliforms, and others.
2. To assess for each vaccine serotype the proportion of children achieving an anti-capsular IgG concentration above the putative protective threshold following the first and second doses of vaccine.
3. To assess the proportion of children achieving opsonophagocytic activity above the putative protective threshold following the first and second doses of vaccine.
4. To determine the incidence of local and systemic reactions following the first and second doses of vaccine.
5. To characterize cellular immune responses in peripheral blood mononuclear cells.
6. To compare carriage and immune responses among children receiving either 1 or 2 doses of PCV10.

## VIII. Design and Methodology

<u>Study design</u>

Observer blinded, randomized, controlled trial

<u>Study site</u>

The study will be conducted in Malindi at the District Hospital and at the Mambrui and Marikebuni dispensaries.

<u>Study populations</u>

Inclusion criteria:

- Age 12-59 months
- Written informed consent

Exclusion criteria:

- Current febrile illness (temperature >38.5°C)

- Previous receipt of any pneumococcal vaccine
- Previous receipt of a DTP-containing vaccine after the 1st year of life
- Previous receipt of hepatitis A vaccine
- Severe malnutrition (mid upper arm circumference <11.5 cm) or other serious medical conditions (e.g., malignancy, AIDS, tuberculosis)
- Convulsions in the past 6 months
- Known allergies to vaccines or vaccine components
- Resident in the Kilifi Demographic Surveillance area
- Intention to leave the study area in the next 6 months

<u>Sample size determination</u>

Given a known background prevalence of nasopharyngeal carriage of the PCV10 serotypes of 25% in this age group, a sample size of 200 in each group would give 90% power (alpha = 0.05) to detect a difference in PCV10-serotype nasopharyngeal colonization prevalence following immunisation of 12% in recipients of 1 or 2 doses of PCV10 and 25% in controls, allowing for 10% attrition.

Studies of PCV10 in children aged <2 years suggest that >95% of vaccinees achieve a seroprotective antibody concentration. With a sample size of 100 vaccinees who received PCV10 at study day 0 and 60, the 95% confidence interval for an observation that 95% achieved the protective antibody threshold would be 89%-98%, allowing for 10% attrition. Similarly, with a sample size of 100 vaccinees aged 12-23 months who received PCV10 at study day 0 and day 60 or 180, the 95% confidence interval for an observation that 95% achieved the protective antibody threshold would be 89%-98%, allowing for 10% attrition.

The sample size will therefore be 600 children (50 children in each of four age groups in each of 3 vaccine programme groups). A subset of 375 children will be followed for immunological evaluation of blood: 50 children aged 12-23 months in each of the three vaccine groups and 25 children in each of the three vaccine groups for the other three age groups. Given this sample size, the study will have 88% power to detect a difference in the proportion of children that develop a seroprotective antibody concentration following one dose of vaccine

of 90%, compared to 99% in those receiving a second dose of vaccine.

Procedures

*Recruitment and randomization*

Participants will be a convenience sample of children aged 12 to 59 months. In collaboration with the Malindi Ministry of Health, notices about the study will be posted at conspicuous locations in the community (e.g., pre-schools and health facilities, with their permission). Interested persons will be asked to telephone the study coordinator for more information or to present to one of the study centres. After obtaining informed consent, children will be assessed for eligibility by study personnel. Medical history and vaccine history will be reviewed. Mid-upper arm circumference will be measured; children with severe malnutrition (MUAC < 11.5 cm) will be referred for admission to hospital.

Equal numbers of eligible children will be admitted into each age group (12-23 months, 24-35 months, 36-47 months, or 48-59 months) and each vaccination group (Table 1). Within each age group children will be sequentially assigned a unique study number corresponding to a computer-generated randomization code that will ensure equal numbers of participants are recruited into the 3 different vaccine groups. As such, there will be 50 children in each age group in each of the three study arms. All children in the 12-23 month age group and the first 25 participants recruited into each of the other three age groups will be enrolled into the immunogenicity sub-study.

Table 1. Vaccination programme

| | Group A | Group B | Group C |
|---|---|---|---|
| Day 0 | PCV10 | PCV10 | Hepatitis A vaccine |
| 2 months | PCV10 | DTaP | DTaP |
| 6 months | DTaP | PCV10 | Hepatitis A vaccine and PCV10 |

*Description of procedures* (Table 2)

The duration of the study is 6-7 months, depending on Study Group. At enrolment, and 4 other visits, an NP swab will be collected. On the first and last study visits, an NP swab will be collected from each nostril. For the subset of children assigned to the immunogenicity study, a sample of blood (5 mL) will be collected from the child's arm at enrolment and at 2 other visits. Costs of study participation (transit fees, missed meals) will be reimbursed.

Table 2. Summary of study activities

| Study Activity | Day 0 | Day of study (acceptable variance) | | | | | | |
|---|---|---|---|---|---|---|---|---|
| | | Day 3 (± 1 d) | Day 30 (± 3 d) | Day 60 (± 3 d) | Day 63 (± 1 d) | Day 90 (± 3 d) | Day 180 (± 7 d) | Day 210 (± 7 d) |
| Consent | X | | | | | | | |
| Eligibility screen[1] | X | | | | | | | |
| Vaccine | X | | | X | | | X | |
| Side effects review | | X | | | X | | | |
| Clinical evaluation | X | X | X | X | X | X | X | X |
| NP Swab | | | | | | | | |
| Group A | X[2] | | X | X | | X | X[2] | |
| Group B | X[2] | | X | X | | | X[2] | |
| Group C | X[2] | | X | X | | X | X[2] | |
| Blood draw[3] | | | | | | | | |
| Group A | X | | X | | | X | | |
| Group B | X | | X | | | | | X |
| Group C | X | | X | | | X | | |

[1]MUAC, temperature, questionnaire.
[2]A swab will be collected from both nostrils.
[3]For children assigned to the immunogenicity subset. See vaccination programme in Table 1.

*Description of the intervention* (Table 2)

All participants will receive a dose of vaccine at the time of enrolment. The study vaccine, Synflorix™ (10-valent pneumococcal vaccine conjugated to protein D of non-typeable *Haemophilus influenzae*) will be manufactured to GMP standards by GlaxoSmithKline, Rixensart, Belgium. Each dose of Synflorix™ contains purified polysaccharides for pneumococcal serotypes 1, 4, 5, 6B, 7F, 9V, 14, 18C, 19F and 23F conjugated to a carrier protein. The comparison vaccines will be commercially available hepatitis A vaccine (HAVRIX™) and DTaP (Infanrix™). Vaccine will be stored between 2°C and 8°C. Vaccines will be transported cold with a temperature sensitive strip and freeze-watch monitor which will indicate whether the vaccine has been stored at temperatures outside the recommended range.

A nurse will administer a 0.5mL intramuscular dose of vaccine into the child's thigh or arm. The left deltoid muscle will be used unless contraindications exist. Study participants/parents will be blinded to the type of vaccination given. Children will be observed for 30 minutes after receipt of vaccine to monitor for any immediate adverse advents. Local and systemic reactions following vaccination will be ascertained during a follow-up visit to the clinic on day 3 following vaccination. Study personnel who are unaware of the randomization status of the participant will make the assessments of reactogenicity. Specific adverse events commonly associated with injectable childhood vaccines will be actively solicited after each vaccine dose. These will include pain, redness, and swelling at the injection site, and fever, irritability, drowsiness, and loss of appetite (Appendix 1). In addition to solicited symptoms, all other adverse events that occur during the study and all concomitant medication use will be recorded on a pro-forma. Parents will be encouraged to bring the child to the district hospital if the child is unwell. The clinical officer will evaluate the child and a standardized safety questionnaire will be administered. The child will be treated appropriately and admitted to hospital, if indicated. All solicited local symptoms will be considered causally related to vaccination. Using their clinical judgment, one of the study investigators will assess the possible causal relationship between vaccination and other reported (non-solicited) adverse events.

Serious adverse events (SAEs) will be reported within 24 hours to the study PI and the local monitor, and the vaccine manufacturers will be informed, as described below. A serious adverse event (SAE) is any untoward medical occurrence that:

a. Results in death.
b. Is life-threatening.

   NB: The term 'life-threatening' in the definition of 'serious' refers to an event in which the subject was at risk of death at the time of the event. It does not refer to an event, which hypothetically might have caused death, had it been more severe.

c. Requires hospitalization or prolongation of existing hospitalization.

   NB: In general, hospitalization signifies that the subject has been admitted at the hospital or emergency ward for observation and/or treatment that would not have been appropriate in the physician's office or out-patient setting. Complications that occur during hospitalization are also considered AEs. If a complication prolongs hospitalization

or fulfils any other serious criteria, the event will also be considered serious. When in doubt as to whether 'hospitalization' occurred or was necessary, the AE should be considered serious.

Hospitalization for elective treatment of a pre-existing condition (known/diagnosed prior to informed consent signature) that did not worsen from baseline is NOT considered an AE.

d. Results in disability/incapacity

NB: The term disability means a substantial disruption of a person's ability to conduct normal life functions. This definition is not intended to include experiences of relatively minor medical significance such as uncomplicated headache, nausea, vomiting, diarrhoea, influenza, and accidental trauma (e.g. sprained ankle) which may interfere or prevent everyday life functions but do not constitute a substantial disruption.

Medical or scientific judgment should be exercised in deciding whether reporting is appropriate in other situations, such as important medical events that may not be immediately life-threatening or result in death or hospitalization but may jeopardize the subject or may require medical or surgical intervention to prevent one of the other outcomes listed in the above definition. These should also be considered serious. Examples of such events are invasive or malignant cancers, intensive treatment in an emergency room or at home for allergic bronchospasm, blood dyscrasias or convulsions that do not result in hospitalization.

Study vaccine related SAEs will be reported to the manufacturer within 24 hours of the study sponsor becoming aware of the event. SAEs not deemed to be study-related will be reported to the manufacturer within 72 hours of the study sponsor becoming aware of the event. SAEs will also be reported to the Ethical Review Committee on a monthly basis.

In the event of a suspected unexpected serious adverse reaction (SUSAR), the subject's study group will be unblinded by the person at CGMR-Coast appointed to hold the randomization code (the ICT department manager) prior to expedited regulatory reporting. Unblinded data will be shared with the vaccine manufacturer within 24 hours of the study sponsor becoming aware of the SUSAR.

After the reactogenicity data have been recorded by study personnel at the post-dose 2 study visit on day 63, the date of the next study visit will be determined according to the participant's study group, with persons in groups A and C returning on day 90 and persons in

group B returning on day 180. The study nurse will disclose the study group to the patient and record the immunizations given on the child's vaccination card on day 180.

Specimen processing and laboratory methods

Blood (5mL) will be collected at the time of enrolment and one month following each dose of vaccine. Samples will be collected in tubes containing sodium heparin and will subsequently be separated into plasma and PBMCs under sterile conditions. A modified enzyme-linked immunosorbent assay (ELISA) will be used to quantitate anticapsular antibody (IgG) to each of the 10 serotypes contained in the vaccine. The laboratory of Dr David Goldblatt at the Institute of Child Health, University College, London, is a designated WHO reference centre for the assay of anti-capsular antibodies and the methods employed have been developed or refined in his laboratory. The ELISA assays will be undertaken in London by staff in the WHO reference laboratory. The functional capacity of antibodies will be analyzed by opsonophagocytosis [25]. Antibody opsonophagocytic activity will be assayed in the GlaxoSmithKline laboratories in Rixensart, Belgium. Aliquots of plasma for ELISA and OPA will be stored in 3.5 mL Sarstedt tubes in a freezer at -80°C until the time of analysis. The phenotype and frequency of T cells and memory B cells will be assessed using fluorochrome-conjugated antibodies and flow-cytometry. The frequency of antigen-specific memory B cells will be determined using ELISPOT [26]. PBMCs will be stored in a liquid nitrogen freezer until the time of analysis.

Nasopharyngeal swabs will be collected prior to and following each dose of vaccine. Swabs will be obtained by passing a Dacron-tipped nasal swab through the nostril, along the floor of the nasal cavity until it touches the posterior nasopharyngeal wall, where it will be left for 2 seconds, rotated 180 and removed. Swabs will be placed in skim-milk tryptone glucose glycerol (STGG) transport media and processed with minimal delay (<6 hours) at the KEMRI CGMR-Coast laboratory, in accordance with WHO recommendations [27]. Following inoculation onto appropriate culture media, swabs will be stored in STGG in a freezer at -80°C. Bacteria will be identified from culture media using standard microbiological/molecular methods. Pneumococci will be identified from gentamicin-blood agar by Optochin susceptibility testing and Quellung serotyping. *Haemophilus influenzae* will be identified from bacitracin-chocolate agar by X and V factor dependence and slide

agglutination serotyping. If necessary, PCR of bacterial isolates will be utilized to confirm identification (e.g., to distinguish non-typeable *Haemophilus influenzae* from *Haemophilus haemolyticus* – done in the GlaxoSmithKline laboratories in Rixensart, Belgium – or to serotype pneumococci – done in the KEMRI-Wellcome Research Laboratories). At the first and last study visit, two NP swabs will be collected. The additional swab will be placed in a sterile 2 mL microcentrifuge tube containing ethanol and stored in a freezer at -80°C prior to analysis. Swabs stored in ethanol will undergo molecular analysis in the United States, using 16S rDNA PCR to characterize the microbial flora. PCR amplicons will be sequenced using a high-throughput sequencer. Microorganisms will be preliminarily identified by BLAST search of the GenBank database. More precise classifications will be made by sequence alignment and phylogenetic reconstruction using bioinformatic analyses and online genetic databases.

Laboratory personnel will be provided with specimens labelled only with study number and will be blinded to the randomization assignment. Assays will be carried out at the KEMRI-Wellcome Research Laboratories, except as specified above. For necessary investigations that are outside the expertise or facilities at KEMRI-Wellcome Research Laboratories, samples may be shipped to collaborating investigators overseas. In such instances, KEMRI-Wellcome investigators will be involved in the analysis and interpretation of data.

Indemnity

Institutional indemnity will be provided by Oxford University. The University has arrangements in place to provide for harm arising from participation in the study for which the University is the Research Sponsor.

Monitor

The Kilifi Clinical Trials Facility monitoring team will be responsible for monitoring the study. A monitoring plan will be developed before the study starts and will include prescheduled visits for the pre-trial, initiation, routine and close-out visits. The study monitor will ensure that entries on CRF are checked against source data and will verify compliance to protocol and SOPs using the standardized procedures established in the facility.

PCV10 safety has been established in large trials of children aged up to 2 years and a similar

safety profile is expected in older children. Additionally, reactogenicity to PCV10 is similar to the PCV7 vaccine, which is widely used in older children. For these reasons, a Data Safety Monitoring Board will not be established.

**IX. Data Management**

Data Storage

Data from the study will be stored in an integrated database run in Filemaker Pro v 9 hosted at the Kilifi CGMR-C server. Twice weekly routine off site back-ups will be conducted. Data access and confidentiality will be overseen by the ICT department manager.

Data Management

Data from the enrolment visit and the reactogenicity follow-up visits will be double entered into an electronic database. Laboratory results will be entered into a local database by laboratory personnel or submitted by electronic file transfer from off-site collaborators, as appropriate. The blinding code will be held by the ICT department manager and will be provided to the PI once she has submitted locked databases.

Data Analysis

Differences in the proportion of nasopharyngeal colonization with pneumococci and non-typeable H *influenzae* between PCV10 recipients and controls will be assessed with the chi-squared test. Pneumococcal serotype-specific IgG will be presented as geometric mean concentrations (GMC). Differences between PCV10 recipients and controls will be tested using a t-test of log-transformed values, as will differences in fold-changes in antibody concentration. Differences between groups in proportion of children with antibody titres and opsonophagocytic activity above the protective thresholds before and after immunization will be assessed with the chi-squared test. Among PCV10 recipients, the proportion of children achieving protective antibody titres and opsonophagocytic activity following first dose of vaccine compared to the second dose will be assessed with the chi-squared test. Paired differences between pre- and post-immunization antibody concentration will be assessed with the Wilcoxon paired signed rank test. An interim analysis will be conducted after study day 90 and results will be provided to GSK and to the Ministry of Health.

**X. Time Frame/Duration of the Project**

Recruitment of study participants will begin in December 2009 and study visits are expected to be complete in October 2010. Laboratory analyses will begin immediately. Microbiological testing of NP swabs and serological testing is expected to be complete in December 2010 and an initial report will be generated at this time. PBMC assays and molecular analysis of NP swab samples will be complete in March 2011. The final report will be written up within 6 months of completion of all laboratory studies.

**XI. Ethical Consideration**

The study has been designed to comply with KEMRI ethical guidelines and with the international ethical guidelines prepared by the Council for International Organizations in Medical Sciences (CIOMS) in collaboration with the WHO. The scientific validity of the study is set out in the Justification and Study Design sections. Initiation of the study will be contingent upon approval from the KEMRI-SCC, the National Ethical Review Committee and the Pharmacy and Poison's Board. This trial will be conducted in compliance with this protocol, the principles of Guidelines for Good Clinical Practice (GCP) and any applicable regulatory requirement(s).

<u>Risks to human subjects</u>

Study activities constitute minimal risk to participants. Collection of blood and sampling of the nasopharyngeal mucous may cause transient discomfort but the procedures carry a negligible risk of harm to the patient. This study involves blinded administration of vaccine, and like all medicines, there may be side effects. The control vaccines, HAVRIX™ and Infanrix™, are licensed for use in children in Kenya. During paediatric trials of HAVRIX™, the following side effects were reported as common: pain, redness, swelling, and fever. During paediatric trials of Infanrix™, the following side effects were reported as common: pain/redness/swelling (10 to 50%), fever (20-30%), drowsiness/irritability/loss of appetite (15-60%). The study vaccine, Synflorix™, is licensed for use in children <2 years of age but will be used "off label" in children aged 24-59 months during this study. Synflorix™ has been studied in children up to 18 months of age and found to be safe. Several studies have confirmed that the reactogenicity profile of Synflorix ™ is comparable to that of PCV7, which

is used in infants and children throughout the world [6]. Side effects common to all vaccines have been reported among infants receiving Synflorix™: redness (46%), pain (31%), swelling (35%), irritability (53%), fever $\geq$38°C (33%), fever >39°C (3%). Among children aged 12 -18 months of age given a booster dose of Synflorix™, general symptoms of grade 3 intensity were reported in 4% and redness >30 mm was reported in 13%. In the proposed study, we shall set up a vigilant system to detect these effects and, if indicated, treat them promptly in accordance with standard treatment protocols.

Synflorix™ has not been licensed or recommended for children in Kenya, nor for children anywhere in the world who are over 2 years of age. At this time it is not known whether children in the intervention arm of the study will be protected from IPD; children in the study may remain at the same risk of IPD as other children throughout Kenya.

Any unexpected side effects or serious adverse events will be reported promptly to the study PI and the local monitor for review, as described above.

Benefits to human subjects

All children in the study will receive the protection against infection offered by the vaccine group to which they are assigned—either PCV10 (possible protection against certain types of pneumonia and meningitis) and DTaP (protection against diphtheria, tetanus, and pertussis) or, PCV10, hepatitis A vaccine, and DTaP. At this time, it is not known whether PCV10 will provide any protection when given to children in the age group targeted by the study; however, PCV10 will be administered to children in all study groups so that any potential benefit is conferred to all study participants.

Families will receive compensation for travel and missed meals. Travel to and from the clinic will be reimbursed at cost. Reimbursement for a missed meal for parent and child will be approximately 100 Kshs per study visit. The exact scheme will be determined in accordance with recommendations from the KEMRI-Wellcome Trust Community Liaison Group and local officials.

Individual informed consent processes

Individual informed consent will be required from the parent/guardian of all study participants. Study personnel will undergo training in the giving of informed consent. This document will be provided in Kiswahili, Kigiriama, and English.

Community engagement strategy**:**

*Community information giving*:

Nursing and medical staff at the study sites will be informed about the study. Communication will be directed to residents of the defined geographic area in which the study is to be conducted. The study project manager will work with the KEMRI communications team to establish the key messages of the study. Key messages will be delivered to relevant groups in consultation with the Community Liaison Group and District Medical Staff.

*Feedback of information*

Information on vaccine reactogenicity and immunogenicity will be communicated to the Head of the Division of Vaccines and Immunizations. Study participants will not be informed of specific test results. Overall results of the study will be communicated to the district medical officer and district public health nurse.

Animal Subjects

None

**XII. Expected Application of the Study and Results**

*General*

The World Heath Organization has recommended that developing countries should incorporate pneumococcal conjugate vaccine into their routine childhood immunization schedules.  Many countries will want to consider a catch-up campaign, as this represents the most rapid way to achieve high levels of vaccine coverage and reductions in rates of disease. However, results of catch-up campaigns will be difficult to interpret without data on the immunological effects of PCV10 in older children. It is anticipated that results of this trial will be useful to public health physicians throughout the developing world.

*Training and capacity building*

This study will help establish links with other public health partners outside of the Kilifi District. Nurses from the local sites will be trained in basic research methods, including administration of informed consent, community engagement, and data reporting. A Kenyan PhD student has been identified to undertake immunological evaluation of PBMCs under the supervision of Dr. Britta Urban. Another student will be sought to undertake molecular analysis of nasopharyngeal swabs under the supervision of Dr. Daniel Frank.

## XIII. References


[1] Berkley JA, Lowe BS, Mwangi I, Williams T, Bauni E, Mwarumba S, et al. Bacteremia among children admitted to a rural hospital in Kenya. N Engl J Med 2005 Jan 6;352(1):39-47.

[2] Brent AJ, Ahmed I, Ndiritu M, Lewa P, Ngetsa C, Lowe B, et al. Incidence of clinically significant bacteraemia in children who present to hospital in Kenya: community-based observational study. Lancet 2006 Feb 11;367(9509):482-8.

[3] CDC. Invasive pneumococcal disease in children 5 years after conjugate vaccine introduction--eight states, 1998-2005. MMWR Morb Mortal Wkly Rep 2008 Feb 15;57(6):144-8.

[4] Poehling KA, Talbot TR, Griffin MR, Craig AS, Whitney CG, Zell E, et al. Invasive pneumococcal disease among infants before and after introduction of pneumococcal conjugate vaccine. JAMA 2006 Apr 12;295(14):1668-74.

[5] Hsu HE, Shutt KA, Moore MR, Beall BW, Bennett NM, Craig AS, et al. Effect of pneumococcal conjugate vaccine on pneumococcal meningitis. N Engl J Med 2009 Jan 15;360(3):244-56.

[6] Vesikari T, Wysocki J, Chevallier B, Karvonen A, Czajka H, Arsene JP, et al. Immunogenicity of the 10-valent pneumococcal non-typeable Haemophilus influenzae protein D conjugate vaccine (PHiD-CV) compared to the licensed 7vCRM vaccine. Pediatr Infect Dis J 2009 Apr;28(4 Suppl):S66-76.

[7] Bermal N, Szenborn L, Chrobot A, Alberto E, Lommel P, Gatchalian S, et al. The 10-valent pneumococcal non-typeable Haemophilus influenzae protein D conjugate vaccine (PHiD-CV) coadministered with DTPw-HBV/Hib and poliovirus vaccines: assessment of immunogenicity. Pediatr Infect Dis J 2009 Apr;28(4 Suppl):S89-96.

[8] Knuf M, Szenborn L, Moro M, Petit C, Bermal N, Bernard L, et al. Immunogenicity of routinely used childhood vaccines when coadministered with the 10-valent pneumococcal non-typeable Haemophilus influenzae protein D conjugate vaccine (PHiD-CV). Pediatr Infect Dis J 2009 Apr;28(4 Suppl):S97-S108.

[9] Wysocki J, Tejedor JC, Grunert D, Konior R, Garcia-Sicilia J, Knuf M, et al. Immunogenicity of the 10-valent pneumococcal non-typeable Haemophilus influenzae protein D conjugate vaccine (PHiD-CV) when coadministered with different neisseria meningitidis serogroup C conjugate vaccines. Pediatr Infect Dis J 2009 Apr;28(4 Suppl):S77-88.

[10] Schuerman L, Prymula R, Chrobok V, Dieussaert I, Poolman J. Kinetics of the immune response following pneumococcal PD conjugate vaccination. Vaccine 2007 Mar 1;25(11):1953-61.

[11] Goldblatt D, Southern J, Ashton L, Richmond P, Burbidge P, Tasevska J, et al. Immunogenicity and boosting after a reduced number of doses of a pneumococcal conjugate vaccine in infants and toddlers. Pediatr Infect Dis J 2006 Apr;25(4):312-9.
[12] Clutterbuck EA, Oh S, Hamaluba M, Westcar S, Beverley PC, Pollard AJ. Serotype-specific and age-dependent generation of pneumococcal polysaccharide-specific memory B-cell and antibody responses to immunization with a pneumococcal conjugate vaccine. Clin Vaccine Immunol 2008 Feb;15(2):182-93.
[13] Dagan R, Givon-Lavi N, Zamir O, Sikuler-Cohen M, Guy L, Janco J, et al. Reduction of nasopharyngeal carriage of Streptococcus pneumoniae after administration of a 9-valent pneumococcal conjugate vaccine to toddlers attending day care centers. J Infect Dis 2002 Apr 1;185(7):927-36.
[14] Hennessy TW, Singleton RJ, Bulkow LR, Bruden DL, Hurlburt DA, Parks D, et al. Impact of heptavalent pneumococcal conjugate vaccine on invasive disease, antimicrobial resistance and colonization in Alaska Natives: progress towards elimination of a health disparity. Vaccine 2005 Dec 1;23(48-49):5464-73.
[15] Mbelle N, Huebner RE, Wasas AD, Kimura A, Chang I, Klugman KP. Immunogenicity and impact on nasopharyngeal carriage of a nonavalent pneumococcal conjugate vaccine. J Infect Dis 1999 Oct;180(4):1171-6.
[16] Hammitt LL, Bruden DL, Butler JC, Baggett HC, Hurlburt DA, Reasonover A, et al. Indirect effect of conjugate vaccine on adult carriage of Streptococcus pneumoniae: an explanation of trends in invasive pneumococcal disease. J Infect Dis 2006 Jun 1;193(11):1487-94.
[17] Ghaffar F, Barton T, Lozano J, Muniz LS, Hicks P, Gan V, et al. Effect of the 7-valent pneumococcal conjugate vaccine on nasopharyngeal colonization by Streptococcus pneumoniae in the first 2 years of life. Clin Infect Dis 2004 Oct 1;39(7):930-8.
[18] O'Brien KL, Millar EV, Zell ER, Bronsdon M, Weatherholtz R, Reid R, et al. Effect of pneumococcal conjugate vaccine on nasopharyngeal colonization among immunized and unimmunized children in a community-randomized trial. J Infect Dis 2007 Oct 15;196(8):1211-20.
[19] Hicks LA, Harrison LH, Flannery B, Hadler JL, Schaffner W, Craig AS, et al. Incidence of pneumococcal disease due to non-pneumococcal conjugate vaccine (PCV7) serotypes in the United States during the era of widespread PCV7 vaccination, 1998-2004. J Infect Dis 2007 Nov 1;196(9):1346-54.
[20] Prymula R, Peeters P, Chrobok V, Kriz P, Novakova E, Kaliskova E, et al. Pneumococcal capsular polysaccharides conjugated to protein D for prevention of acute otitis media caused by both Streptococcus pneumoniae and non-typable Haemophilus influenzae: a randomised double-blind efficacy study. Lancet 2006 Mar 4;367(9512):740-8.
[21] Bogaert D, van Belkum A, Sluijter M, Luijendijk A, de Groot R, Rumke HC, et al. Colonisation by Streptococcus pneumoniae and Staphylococcus aureus in healthy children. Lancet 2004 Jun 5;363(9424):1871-2.
[22] Regev-Yochay G, Dagan R, Raz M, Carmeli Y, Shainberg B, Derazne E, et al. Association between carriage of Streptococcus pneumoniae and Staphylococcus aureus in Children. Jama 2004 Aug 11;292(6):716-20.
[23] Frank DN, Spiegelman GB, Davis W, Wagner E, Lyons E, Pace NR. Culture-independent molecular analysis of microbial constituents of the healthy human outer ear. J Clin Microbiol 2003 Jan;41(1):295-303.

[24] Fiore AE, Wasley A, Bell BP. Prevention of hepatitis A through active or passive immunization: recommendations of the Advisory Committee on Immunization Practices (ACIP). MMWR Recomm Rep 2006 May 19;55(RR-7):1-23.
[25] Burton RL, Nahm MH. Development and validation of a fourfold multiplexed opsonization assay (MOPA4) for pneumococcal antibodies. Clin Vaccine Immunol 2006 Sep;13(9):1004-9.
[26] Crotty S, Aubert RD, Glidewell J, Ahmed R. Tracking human antigen-specific memory B cells: a sensitive and generalized ELISPOT system. J Immunol Methods 2004 Mar;286(1-2):111-22.
[27] O'Brien KL, Nohynek H. Report from a WHO Working Group: standard method for detecting upper respiratory carriage of Streptococcus pneumoniae. Pediatr Infect Dis J 2003 Feb;22(2):e1-11.

## XIV. Appendices

1) Role of each investigator

| | |
|---|---|
| Mahfudh Bashraheil | Clinical Trials Facility – study coordinator |
| Morris Buni | Medical Superindendent, Malindi – study design and coordination |
| Roma Chilengi | Head Clinical Trials Facility – study design, analysis |
| Daniel Frank | Molecular biologist – supervision of molecular analysis of swabs |
| David Goldblatt | Immunologist – measurement of immunogenicity |
| Fauzat Hamid | Clinical Officer – SAE detection and management |
| Laura Hammitt | Principal investigator – overall study coordination |
| Ali Hussein | DMOH, Malindi – study design and coordination |
| Tatu Kamau | Head DVI – vaccine policy |
| Susan Morpeth | Microbiologist – supervision of microbiological analysis of swabs |
| Daniel Muli | PhD student working on T and B cell immunology studies |
| Anthony Scott | Co-principal investigator – study design, funding, analysis |
| Benjamin Tsofa | KEMRI-MOH liaison |
| Britta Urban | Immunologist –supervision of T and B cell studies |

2) Reactogenicity assessment tool

**Reactogenicity assessment tool**

| | Day of vaccine | | Day 1 after vaccine | | Day 2 after vaccine | | Day 3 after vaccine | |
|---|---|---|---|---|---|---|---|---|
| | | | | | | | | |
| **Local** | | | | | | | | |
| Pain, any | Y | N | Y | N | Y | N | Y | N |
| Pain, grade 2[1] | Y | N | Y | N | Y | N | Y | N |
| Pain, grade 3[2] | Y | N | Y | N | Y | N | Y | N |
| Redness, any | Y | N | Y | N | Y | N | Y | N |
| Redness, >30 mm | Y | N | Y | N | Y | N | Y | N |
| Swelling, any | Y | N | Y | N | Y | N | Y | N |
| Swelling, >30 mm | Y | N | Y | N | Y | N | Y | N |
| | | | | | | | | |
| **General** | | | | | | | | |
| Fever (felt warm) | Y | N | Y | N | Y | N | Y | N |
| Drowsiness, any | Y | N | Y | N | Y | N | Y | N |
| Drowsiness, grade 2[1] | Y | N | Y | N | Y | N | Y | N |
| Drowsiness, grade 3[2] | Y | N | Y | N | Y | N | Y | N |
| Irritability, any | Y | N | Y | N | Y | N | Y | N |
| Irritability, grade 2[1] | Y | N | Y | N | Y | N | Y | N |
| Irritability, grade 3[2] | Y | N | Y | N | Y | N | Y | N |
| Loss of appetite, any | Y | N | Y | N | Y | N | Y | N |
| Loss of appetite, grade 2[1] | Y | N | Y | N | Y | N | Y | N |
| Loss of appetite, grade 3[2] | Y | N | Y | N | Y | N | Y | N |
| Other | | | | | | | | |

[1]Grade 2: pain defined as cried/protested on touch; drowsiness defined as interfered with normal daily activities; irritability/fussiness defined as crying more than usual/interfered with normal daily activities; loss of appetite defined as eating less than usual/interfered with normal daily activities.
[2]Grade 3: pain defined as cried when limb was moved/spontaneously painful; drowsiness defined as prevented normal daily activities; irritability/fussiness defined as crying that could not be comforted/prevented normal daily activities; loss of appetite defined as no eating at all.
